# Hydrophilic Poly(Iminopyridinium Ylide)s: Defining a New Chemical Space for Poly(ylide)s

**DOI:** 10.1002/marc.202500641

**Published:** 2025-10-14

**Authors:** Noël René Schneider, Aleksandra M. Orlova, Nuwanthika Dilrukshi Kumarage, Patrick Théato, Kevin Neumann

**Affiliations:** ^1^ Institute for Molecules and Materials Radboud University Nijmegen The Netherlands; ^2^ Soft Matter Synthesis Laboratory – Institute for Biological Interfaces III (IGB‐3) Karlsruhe Institute of Technology Karlsruhe Germany; ^3^ Institute for Chemical Technology and Chemistry (ITCP) Karlsruhe Institute of Technology Karlsruhe Germany

**Keywords:** biomolecules, nanomedicine, poly(iminopyridinium ylide)s, poly(ylide), post‐polymerization modification, zwitterionic polymers

## Abstract

Rising hypersensitivity to PEG and accelerated blood clearance highlight the need for alternative charge‐neutral hydrophilic polymers. Poly(ylide)s represent a class of hydrophilic polymers with biocompatibility and antifouling properties. Here, we explore poly(iminopyridinium ylide) (PIPY) as a versatile nano‐ and biomedical building block. PIPY is synthesized via post‐polymerization modification of poly(pentafluorophenyl acrylate), maintaining a narrow molecular weight distribution. PIPY's structure was confirmed by NMR, FTIR, and SEC. PIPY is soluble in water, saline, MeOH, and DMSO, and remains stable from strongly acidic to physiological pH. Critical aggregation concentration and DOSY NMR measurements indicate an anti‐polyelectrolyte effect and minimal responsiveness to apolar environments, respectively. Notably, PIPY exhibits minimal, entropically driven binding to biomolecules such as bovine serum albumin and lysozyme. This low interaction is critical for its ability to prevent insulin fibrillation upon heating, suggesting utility as a protein‐stabilizing matrix. These combined properties position PIPY as a promising material for future bio‐ and nanomedical applications.

## Graphical abstract

Poly(iminopyridinium ylide) (PIPY) is reported as an overall charge‐neutral hydrophilic polymer with good water‐solubility, stability across physiologically relevant pH levels, and minimal biomolecular interactions. PIPY shows prevention of insulin fibrillation, which highlights its protein‐stabilizing capacity. Synthesized via post‐polymerization modification, PIPY emerges as a versatile polymer platform for nanomedical and biomedical applications.

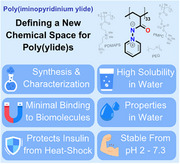

## 1 Introduction

Charge‐neutral hydrophilic polymers have been established as one of the most important building blocks for functional materials, in particular for bio‐ and nanomedical applications [1]. Currently, poly(ethylene glycol) (PEG) is considered the “gold standard” among charge‐neutral hydrophilic polymers and finds application in a variety of areas, such as drug formulations, polymer‐conjugates and materials [2, 3]. For example, PEGylation, i.e., the process of attaching PEG chains to a chemical substrate, such as nanoparticle‐encapsulated hydrophobic or sensitive drugs, allows their administration under physiological conditions and may prevent premature release and degradation [4]. Notably, this concept was instrumental in the development of the COVID‐19 mRNA vaccines by Pfizer and Moderna [5]. PEGylated surfaces display a significantly decreased protein adsorption, which in turn decreases their clearance from blood, thereby increasing the blood circulation time. This effect is commonly referred to as a ’stealth property’ [6, 7]. PEG also finds application as an excipient in liquid or solid drug formulations containing proteins, enzymes and/or (m)RNA due to its capability to protect these biomolecules from degradation by external stress‐factors including heat [8, 9, 10]. Despite this success, the search for PEG alternatives has become increasingly critical. This need arises because of the rising occurrence of hypersensitive reactions to PEG among people, which is caused by anti‐PEG antibodies due to its excessive application in pharmaceutics, cosmetics and food production [11]. Additionally, the accelerated blood clearance (ABC) phenomenon, referring to the increased clearance and decreased efficacy of PEGylated drugs upon multiple administrations, poses a significant challenge for existing and future approaches using PEG as a stealth agent [5, 12].

Zwitterionic polymers have been established as a promising PEG‐alternative more than a decade ago [13]. A shared feature of all zwitterionic polymers is the (near‐to) equimolar balance of positively and negatively charged groups within these structures, resulting in a variety of properties like their overall charge‐neutrality and commonly high solubility in water [14]. Typically, zwitterionic polymers are classified as either polyampholytes or polybetaines. Polyampholytes are defined in the context of this article to be obtained by copolymerization of two monomers, one of which contains a negatively charged subunit, and the other one containing a positively charged subunit [15]. As it is effectively impossible to achieve a perfect 50/50 incorporation ratio between charges, polyampholytes are also commonly referred to as pseudo‐zwitterionic polymers. In contrast, polybetaines contain a positive and negative charge in the side group of each constitutional unit, meaning that they achieve perfect charge‐neutrality. It has been shown that these types of polymers display excellent antifouling properties, prolonged lifetime in the bloodstream, as well as capabilities to protect proteins from heat‐induced degradation [16, 17, 18, 19, 20].

More recently, poly(ylide)s were established as a new class of charge‐neutral polymers exhibiting a strong zwitterionic character and pronounced hydrophilicity [21]. Their inherent minimal opposite‐charge separation significantly decreases the dipole moment relative to other zwitterionic structures, while still showing high solubility in polar solvents as well as antifouling properties [22]. Recent studies by Sarker and coworkers on the importance of a small dipole moment for preventing interactions with biomolecules via dipole/dipole interactions, as well as Sánchez‐Cerrillo's studies on poly(ylidic) termini for PEG to reduce antigenicity, showcase the general capabilities of poly(ylide)s as nanomedical building blocks [23, 24]. Despite that, hydrophilic poly(ylide)s remain surprisingly underexplored, with only a handful of classes reported to date, including poly(N‐oxide)s, poly(sulfur ylide)s, poly(phosphorus ylide)s, and poly(imino phosphorane)s [25, 26, 27, 28, 29]. The contrast between the significant potential and current gaps in understanding their properties highlights the need for more fundamental research toward the synthesis, design and applications of poly(ylide)s. In this context, iminopyridinium ylides have been mostly unaccounted for, despite preliminary studies dating back more than a century ago [30, 31]. More detailed investigations on these compounds were provided by Snieckus and Kan in 1970, who focused on the reactivity of the iminopyridinium ylide N─N bond as a structural motif that distinguishes it from other ylides [32]. Then, in 1983 and 1988, Kondo and Taylor reported on the synthesis of a variety of polymeric (imino)pyridinium ylides via the reaction of isocyanatoethyl methacrylate with 1‐aminopyridinium ylide and the reaction of poly(4‐vinylpyridine) with ethylazidoformate, yielding an in‐situ nitrene, respectively [33, 34]. Expanding on these studies around two decades later, Klinger et al. noted the successful synthesis of poly(iminopyridinium ylide) (PIPY) via post‐polymerization modification (PPM) of poly(pentafluorophenyl acrylate) (PPFPA) [35]. Apart from its reactivity, other properties, especially as a hydrophilic polymer in a biomedical context, have remained elusive so far.

Therefore, the synthesis and evaluation of PIPY was targeted in this study to expand the toolbox of hydrophilic polymeric ylides with far‐reaching potential for applications as a building block in bio‐ and nanomedical contexts. In this work, we report on the synthesis of PIPY via PPM of PPFPA, which was prepared by reversible addition–fragmentation chain‐transfer (RAFT) polymerization, with 1‐aminopyridinium iodide (1‐API). The resulting polymer was characterized by nuclear magnetic resonance (NMR) spectroscopy, size exclusion chromatography (SEC), and Fourier‐transform infrared (FTIR) spectroscopy. The behavior of PIPY in aqueous environments was systematically investigated, including its chemical stability at various pH values, aggregation properties via fluorescence spectroscopy and diffusion ordered spectroscopy (DOSY) NMR, as well as its interactions with biomolecules using isothermal titration calorimetry (ITC). The behavior of PIPY in water was compared with that of commercially available hydrophilic PEG, as well as with two synthetically accessible polybetaines: poly([2‐(methacryloyloxy)ethyl]dimethyl‐(3‐sulfopropyl)ammonium hydroxide) (PDMAPS) and poly(2‐methacryloyloxyethyl phosphorylcholine) (PMPC). We believe that this work serves as a foundation to gain further fundamental understanding of the properties of poly(ylide)s, thereby guiding future research endeavors.

## 2 Results and Discussion

### 2.1 Synthesis and Physicochemical Properties of PIPY

For the synthesis of PIPY, a post‐polymerization modification approach was chosen, based on the reaction of the activated ester polymer, PPFPA, via substitution with the amino‐functionalized pyridinium salt 1‐aminopyridinium iodide (1‐API). The salt was synthesized by the reaction of pyridine with hydroxy‐o‐sulfonic acid, followed by treatment with hydroiodic acid (HI). In the first step to afford the desired polymer, the pentafluorophenyl acrylate (PFPA) monomer was synthesized according to a previously reported procedure [36]. A controlled radical polymerization was selected for the synthesis of PPFPA to enable precise control over the molecular weight and dispersity of the resulting polymer precursor. In terms of nano‐ and biomedical applications, the RAFT approach also enables the future installation of reactive (click) handles applicable for bioconjugation and diversification by leveraging the synthetic capabilities of the terminal trithiocarbonate groups [37, 38, 39]. For this, RAFT polymerization of PFPA was carried out in 1,4‐dioxane using AIBN as the initiator and 2‐(dodecylthiocarbonothioylthio)‐2‐methylpropionic acid (DDMAT) as the chain transfer agent. The chemical structure of the resulting active ester polymer was confirmed by ^1^H and ^19^F NMR (Figures SF7 and SF8, Supporting Information) and FTIR‐spectroscopy (Figure SF27, Supporting Information). The polymer precursor exhibited a narrow, unimodal molecular weight distribution with a Đ = 1.48 and values of *M_n_* = 8.2 kg mol^−1^ and *M_w_* = 12 kg mol^−1^, as determined by SEC in THF using PS‐standard (Figure SF30, Supporting Information).

**FIGURE 1:**
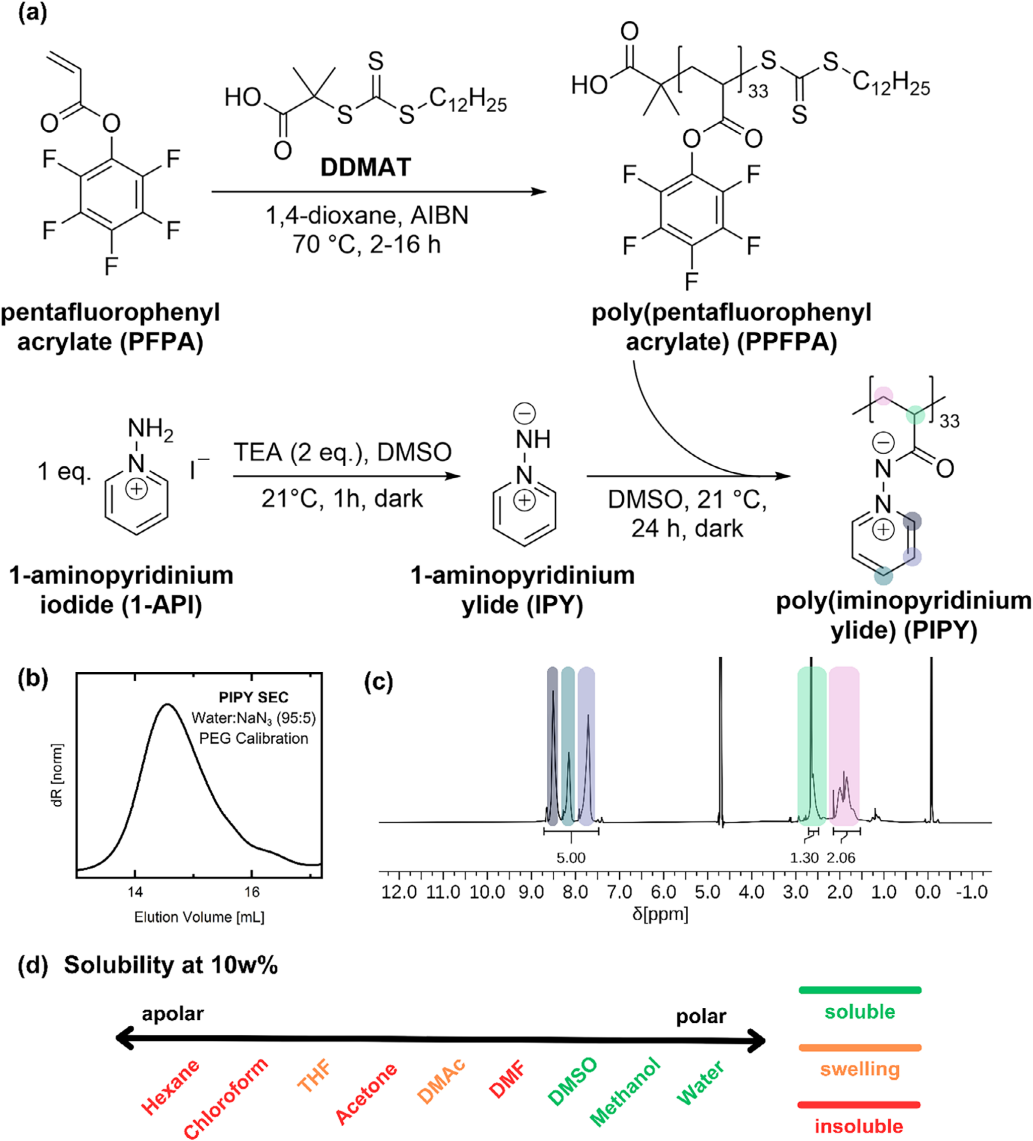
(a) Reaction scheme for the synthesis of PIPY via post‐polymerization modification of RAFT‐polymerized PPFPA with 1‐API in DMSO; (b) SEC trace of PIPY measured in water with 0.05% NaN_3_ buffer solution (PEG standard); (c) ^1^H NMR spectrum of PIPY recorded in DMSO‐d_6_; (d) Solubility test of PIPY (10 wt%) in various solvents.

Subsequently, the active ester groups of PPFPA were quantitatively converted into iminopyridinium ylides by reaction with 1‐API using the PPM approach as reported previously by Klinger et al. [35]. This reaction was performed in DMSO, allowing efficient substitution of the activated ester groups with the amino‐functionalized pyridinium ylide, yielding the target polymer PIPY. In the first step of the PPM, 1‐API was deprotonated by reaction with triethylamine (TEA) in DMSO for 1 h in the dark to generate the reactive iminopyridinium ylide (IPY). In the second step, this reaction mixture was combined with a solution of PPFPA in DMSO and stirred at room temperature (RT) for 24 h. The synthetic route to PIPY is shown in Figure 1a.

Complete conversion of the activated ester groups was confirmed by ^19^F NMR spectroscopy of PIPY, which showed the complete disappearance of pentafluorophenyl signals after PPM (Figure SF11, Supporting Information). The ^1^H NMR spectrum of the resulting polymer revealed the appearance of broad signals corresponding to the aromatic protons of the pyridinium fragment in the region of 8.60–7.10 ppm (Figure 1c), further supporting successful functionalization. In addition, FTIR spectroscopy showed the complete disappearance of the characteristic ester carbonyl absorption band at 1782 cm^−1^ and the aromatic C═C stretching vibrations, influenced by electron‐withdrawing fluorine substituents at 1520 cm^−1^, indicating full substitution of the activated ester moieties (Figure SF29, Supporting Information).

Molecular weights of PIPY were evaluated by aqueous SEC using PEG standards (Figure 1b). The SEC trace exhibited a unimodal distribution with no observable change in dispersity (Đ = 1.38) (Figure SF31, Supporting Information), confirming that the PPM proceeded without side reactions. The use of aqueous SEC was dictated by the solubility profile of the modified polymer. Prior to modification, PPFPA exhibited hydrophobic behavior and was insoluble in water, but soluble in a broad range of organic solvents. In contrast, after PPM, PIPY displayed a hydrophilic character, with solubility limited to highly polar solvents such as methanol and water. This enhanced hydrophilicity is attributed to the zwitterionic nature of the repeating units in PIPY, which contain both positively and negatively charged nitrogen atoms, enabling classification of the polymer as a zwitterionic material.

**FIGURE 2:**
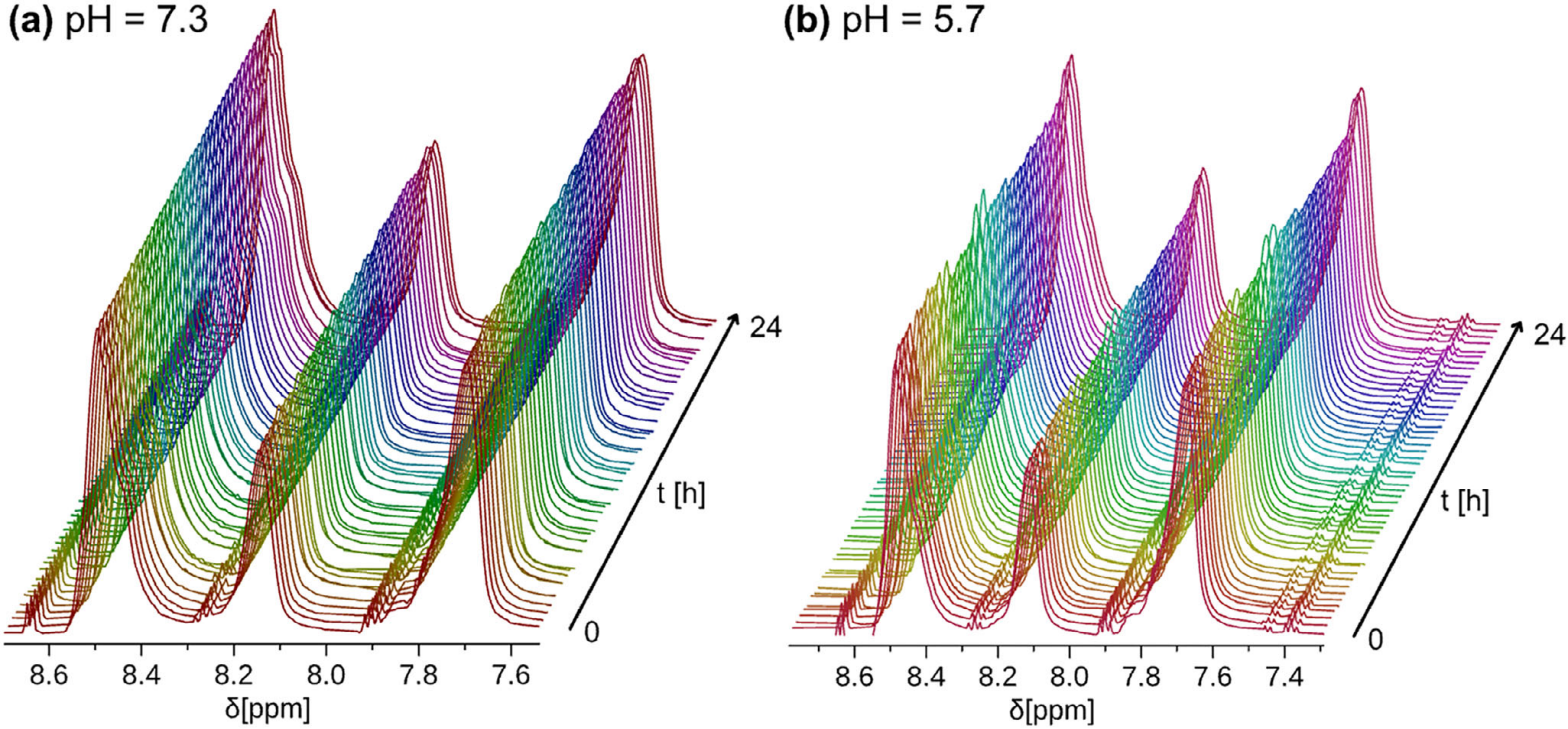
^1^H NMR spectra of PIPY recorded over 24 h at (a) pH 7.3 and (b) pH 5.7 as a stability test under physiologically relevant conditions.

Next, the stability of PIPY toward chemical transformation and degradation under daylight in aqueous medium was evaluated. Literature reports indicate that upon UV irradiation (≈320 nm), PIPY may undergo photoisomerization resulting either in a ring expansion to yield hydrophobic 1,2‐diazepines via an excited singlet state, or alternatively in a N─N bond cleavage via a triplet state with release of pyridine [32, 35, 40]. However, according to ^1^H NMR analysis (Figure SF16, Supporting Information), no noticeable changes in the chemical structure were observed after PIPY was exposed to daylight for 7 h. This stability under ambient light conditions suggested that energy‐rich UV light is necessary to induce the photoisomerization. In addition, polymer stability across varying pH values was investigated as a critical factor for application under physiological conditions. To assess whether PIPY is acid‐responsive, we recorded ^1^H NMR spectra over the course of one day at pH values of 7.3 and 5.7, mimicking healthy and tumorous tissue respectively (Figure 2) [41, 42]. In both cases, we did not observe the appearance of signals corresponding to either the aromatic protons of the diazepine ring or the free pyridine protons. PIPY showed no significant changes in signal intensity over this extended period of time, indicating a reasonable stability at both pH values.

Both samples were stored at pH 7.3 and pH 5.7 at 4°C over the course of 34 days in order to determine whether PIPY might degrade during prolonged storage. The obtained spectra (Figures SF14 and SF15, Supporting Information) did not show the appearance of any new signals nor an increase in the ratio between the small molecule peaks and polymer signals. Thus, it was concluded that PIPY does not degrade under physiologically relevant pH values, underlining its suitability as a biomaterial. Finally, PIPY was dissolved in deuterium oxide (D_2_O) and exposed to deuterium chloride (DCl), resulting in a pH < 2. Analysis by ^1^H NMR once more revealed no sign of polymer degradation (Figure SF17, Supporting Information), i.e., suggesting a stability of PIPY under acidic conditions.

### 2.2 Behavior in Aqueous Environment

**FIGURE 3:**
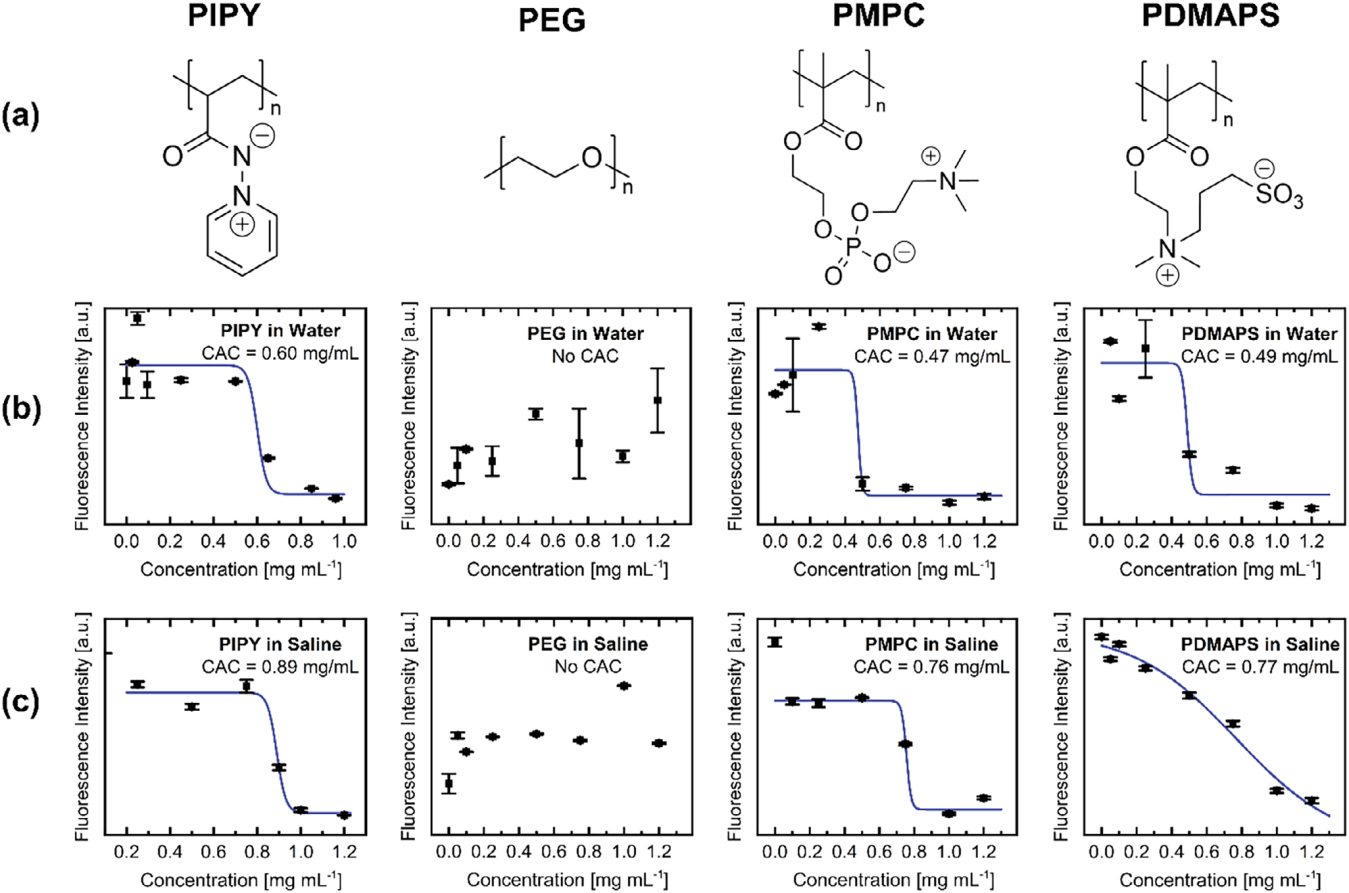
(a) Chemical structures of the polymers investigated in this work: PIPY, PEG, PMPC and PDMAPS. (b) The critical aggregation concentrations (CACs) of PIPY, PEG, PMPC and PDMAPS in water and (c) in saline (1 m).

Given the stability of PIPY in aqueous solutions at different pH‐values, its aggregation behavior in aqueous systems was further investigated. Self‐aggregation can significantly influence key factors such as surface tension, solubility, and the polymer's interactions with biomolecules [43, 44]. To that aim, the following experiments were conducted on PIPY, commercially available PEG (5 kDa), as well as two synthetically accessible polybetaines with inversed charge orientation, namely PDMAPS and PMPC (Figure 3; Sections S2.1 and S2.2 and Figures SF12 and SF13, Supporting Information). Positioning PIPY alongside PEG, PDMAPS, and PMPC allows for a more precise definition of its properties in the context of hydrophilic and biocompatible polymers [45, 46, 47, 48].

First, critical aggregation concentrations (CAC) of all four polymers were assessed (Figure 3 and Table 1). Despite the similarity in naming to the critical micelle concentration (CMC), we chose this nomenclature for a more appropriate discussion as it is not expected that the polymers form well‐defined micelles because of the lack of defined hydrophobic segments [49]. Here, CACs were assessed using fluorescence spectroscopy with Rhodamine 6G. The selection of the fluorophore was based on its absorption maximum in the visible region (λ_ex_ = 530 nm in water), in contrast to PIPY, which absorbs in the UV region (≈320 nm) [48, 50]. In the same instance, the CAC measurements can also provide insights into the polymers’ responsiveness to elevated salt concentrations that are commonly found under physiological conditions [51]. For charge‐containing polymers, two effects are of relevance, namely the so‐called polyelectrolyte and the anti‐polyelectrolyte effects. The polyelectrolyte effect refers to a decrease in solubility upon increased salt concentration [52]. This effect is mainly relevant for polyelectrolytes that contain only one type of charge per side‐chain. In aqueous environments, these polymers attain an extended conformation due to favorable ion‐dipole interactions with water and charge‐repulsion between side‐chains. In consequence, salt increases the ionic strength of the solvent, making solvent‐polymer repulsion a stronger factor compared to the intramolecular repulsion, leading to the polymer coiling up and, eventually, salting out. However, zwitterionic polymers with positive and negative charges per side‐chain display an anti‐polyelectrolyte effect [53]. For example, the solubility of the polymer increases with increasing concentrations of salt. Because of the presence of opposite charges in the side‐groups, they tend to form aggregates due to intra‐chain electrostatic attraction. The addition of salt makes a more extended structure more favorable, thereby increasing the solubility. In this context, a decrease of the CAC upon the addition of salt suggests a polyelectrolyte effect, whereas an increase in the CAC points toward an anti‐polyelectrolyte effect [49, 53].

**TABLE 1:** The critical aggregation concentrations (CACs), the slope of the sigmoidal approximation (k) and Hill coefficient (n) of PIPY, PEG, PMPC, and PDMAPS in water and 1 m saline. CACs were determined by fluorescence by fluorescence spectroscopy using Rhodamine 6G. Python scripts (Section S5, Supporting Information) were used to fit the data, derive the slope (k) and calculate the Hill coefficient (n) to assess cooperativity.

	Water			Saline (1 m)		
Polymer	CAC [mg mL^−1^]	*K* [mL mg ^−1^]	*n*	CAC [mg mL^−1^]	*K* [mL mg ^−1^]	*n*
PIPY	0.60	−18.6	−0.98	0.89	−47.8	−0.73
PEG	N/A	N/A	N/A	N/A	N/A	N/A
PMPC	0.47	−90.8	−0.58	0.76	−74.3	−0.83
PDMAPS	0.49	−69.9	−1.10	0.77	−3.4	−1.68

In good agreement with the literature, PEG did not show self‐aggregation at concentrations below 1 mg mL^−1^ in either water or saline [54]. However, PIPY, PMPC and PDMAPS all displayed a distinct CAC of 0.60/0.89, 0.47/0.76, and 0.49/0.77 mg mL^−1^ in water/saline, respectively (Figure 3 and Table 1). Using 1 m saline as the solvent, all three polymers exhibited a pronounced anti‐polyelectrolyte effect with substantially increased CAC values compared to water. Notably, in both water and saline, PIPY consistently showed the highest CAC values.

In addition to CACs, the slope of the sigmoidal approximation, *k* (Sections S3.2 and S5, Supporting Information), is reported to indicate possible trends in aggregation cooperativity [55]. For PIPY, an increased slope in saline relative to water was determined, which was opposite to the trend of both polybetaines. An increase in slope may indicate an enhanced cooperativity for aggregation in the case of PIPY in saline. Fitting the measurement data to the Hill model delivered the Hill coefficient *n* (Table 1) [56]. For all polymers, the Hill coefficient was below 0, indicating an overall noncooperative type of aggregation. In the case of PIPY, *n* slightly increased in saline, but in contrast decreased for PMPC and PDMAPS. I.e., the aggregation of PIPY appeared to be more cooperative in saline than in water, but less cooperative for PMPC and PDMAPS.

To gain further insights into the nature of the observed self‐aggregation, we also conducted diffusion‐ordered spectroscopy (DOSY) NMR experiments at polymer concentrations of 5 mg mL^−1^, i.e., significantly above the CAC, which also enabled sufficient signal intensity (Table 2; Figures SF18–SF25, Supporting Information). The experimentally determined diffusion constants were used to approximate the polymers’ hydrodynamic radii with the Stokes‐Einstein equation (Equation 1) [57]. For all DOSY NMR experiments, a double stimulated echo experiment with three spoil gradients for convection compensation (“dstebpgp3s”) was chosen because of its better suitability for polymeric samples [58].

(1) $$R_{H}=\frac{kT}{6\pi \eta D}$$

**TABLE 2:** Diffusion coefficients (*D*) of PIPY, PEG, PMPC and PDMAPS, measured by DOSY NMR in D_2_O and MeOD. The hydrodynamic radii (*R*_H_) were calculated using the Stokes–Einstein equation (Equation 1). Standard Deviations for *D* were derived from the differences in diffusion constants belonging to the same species.

Polymer	*D* [10^−10^ m^2^ s^−1^] in water	*R*_H_ [nm] in water	*D* [10^−10^ m^2^ s^−1^] in methanol	*R*_H_ [nm] in methanol
**PIPY**	1.70 ± 0.02	1.15	2.55 ± 0.06	1.48
**PEG**	1.71 ± 0.20	1.15	3.14 ± 0.14	1.20
**PMPC**	1.27 ± 0.05	1.54	1.81 ± 0.04	2.09
**PDMAPS**	2.40 ± 0.38	0.82	12.5 ± 1.5	0.30

The Stokes–Einstein equation [57] was used to approximate the polymers’ hydrodynamic radii using the diffusion constants determined by DOSY NMR (Table 3). Here, k = 1.38 * 10^−23^ m^2^ kg s^−2^ K^−1^, *T* = 298 K, η(D_2_O) = 1.095 mPa s [59], η(methanol‐d4) = 0.569 mPa s [60].

**TABLE 3:** Zeta potential measurements of PIPY, PEG, PMPC, and PDMAPS at a concentration of 2 mg mL^−1^ in Milli‐Q water.

Polymer	ζ‐potential [mV]
PIPY	−3.6
PEG	−2.2
PMPC	−9.9
PDMAPS	−16.3

The measurements revealed a single diffusion coefficient, and hence indicated a single diffusing species, for each polymer in both tested solvents. Since intermolecular aggregation is an equilibrium process, DOSY NMR should reveal at least two different diffusing species, one corresponding to the aggregate and one to the free polymer [61, 62]. Since this was not the case here, it may suggest that the observed CACs rather relate to intramolecular folding in the form of conformational rearrangements or a change in hydration shell and that PIPY, PEG, PMPC and PDMAPS exist as singular polymer species in solution. This conclusion goes along with the calculated hydrodynamic radii whose size (<3 nm) corresponds to single polymer chains rather than intermolecular aggregates [57]. In MeOD, PIPY, PEG and PMPC all showed an increase in their respective hydrodynamic radius, with PIPY and PEG showing only moderate changes compared to PMPC. In contrast, PDMAPS is the only tested polymer that showed a decrease in *R_H_* upon dissolution in methanol, i.e., indicating an opposite trend in direct comparison with the charge‐inversed polybetaine PMPC. We have recently demonstrated that the dipole moment of sulfur ylides is solvent‐dependent, which may explain why methanol serves as a more effective solvent for PIPY, but, for example, less effective for PDMAPS [63]. Generally, polybetaines are considered to be highly responsive to their environments, which make them accessible for various applications, but therefore also less broadly applicable compared to the ‘gold‐standard’ PEG [64, 65]. In this context, PIPY's more moderate change in *R_H_*, especially relative to PMPC, seems to thus make it more similar to PEG, which is particularly interesting toward the aim of finding suitable alternatives to this polymer. More detailed investigations into the nature of the intramolecular folding and the underlying driving forces are required to gain better insights into this phenomenon and the differences between polymers.

Overall, PIPY shows comparable properties in terms of intramolecular folding to established PMPC and PDMAPS in both water and saline. However, its behavior in more apolar environments seems to parallel PEG rather than these typical polybetaines. This diversity in properties therefore offers numerous handles to fine‐tune PIPY by, e.g., co‐ and/or sequence‐defined polymerization to access so far less explored spaces in targeted property development of polymeric materials for bio‐ and nanomedical applications.

### 2.3 Interaction with Biomolecules

**FIGURE 4:**
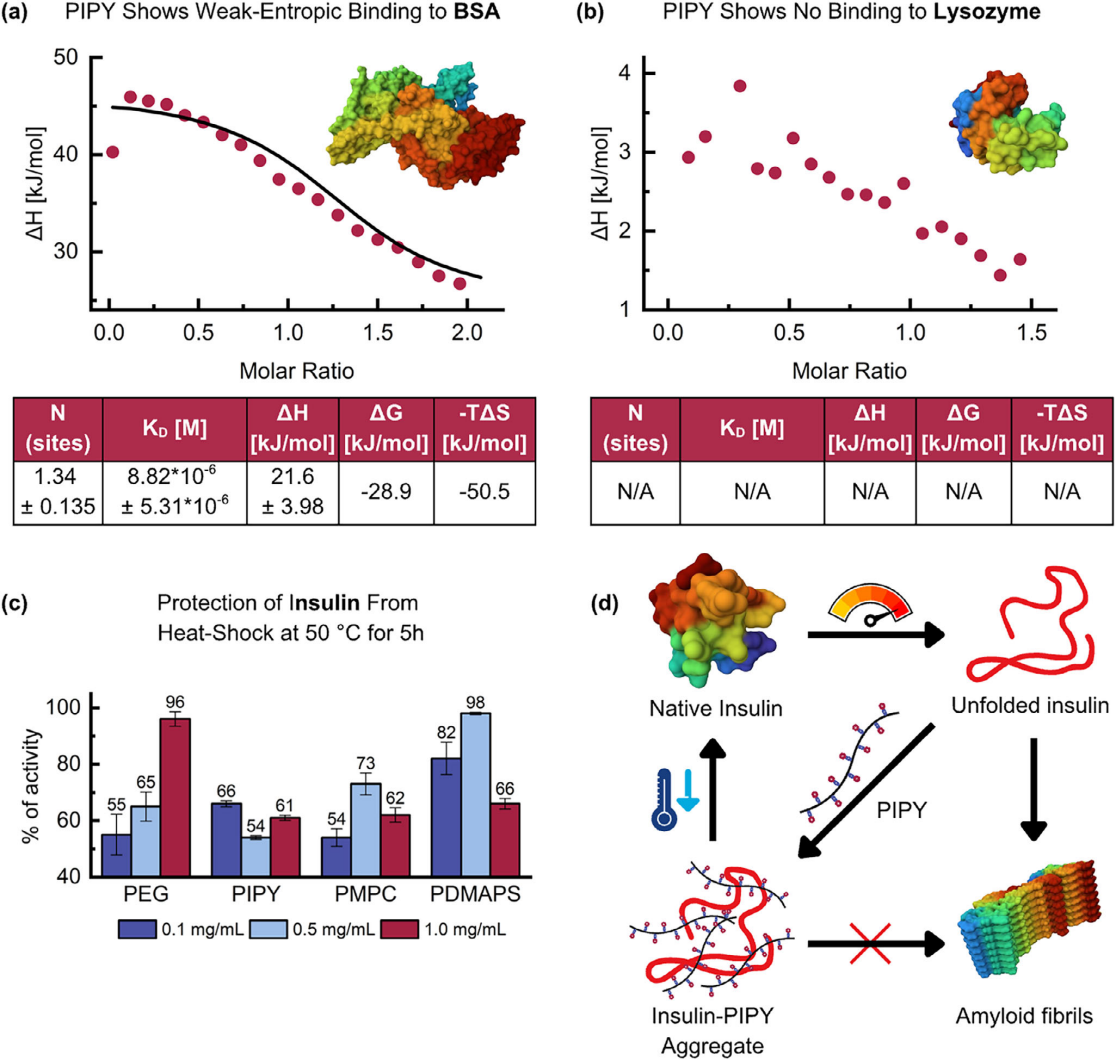
Isothermal Titration Calorimetry (ITC) results of PIPY with (a) BSA and (b) LYZ; (c) Retained insulin activity after heating for 5 h at 50°C in the presence of PEG, PIPY, PMPC and PDMAPS relative to unheated insulin (100% activity) and heated insulin without excipients (0% activity) according to ThT assays; (d) A schematic drawing of how PIPY and other hydrophilic polymers can prevent insulin from forming amyloid fibrils upon heating according to Rajan et al. [18, 76]. Figures of the proteins BSA (PDB ID: 3V03), LYZ (PDB‐ID: 1DPX), insulin (PDB‐ID: 1TRZ) and insulin amyloid fibrils (PDB‐ID: 8SBD) were created using Mol* [77].

Zwitterionic polymers are of particular interest for bio‐ and nanomedical applications because of their hydrophilicity and weak interactions with biomolecules, among others. Here, we explore properties of PIPY relevant in this context to broaden the established chemical space toward this field. First, ITC of PIPY with bovine serum albumin (BSA) and lysozyme (LYZ) in phosphate‐buffered saline (PBS) buffer (pH 7.4) was performed (Figure 4a,b). With an isoelectric point (pI) of 4.7 and 11 for BSA and LYZ, respectively, BSA attains a net negative and LYZ a net positive charge at the given pH [66, 67]. The results showed that PIPY entropically binds to BSA, while no binding to LYZ was detected. At first, these results may look counterintuitive as PIPY, according to zeta potential measurements (Table 3; Figures SF32–SF35, Supporting Information), exhibited a net negative surface charge and should therefore preferentially bind to the net positively charged lysozyme, and repel the net negatively charged BSA. Indeed, the enthalpy of binding between PIPY and BSA was determined as 21.6 ± 3.98 kJ mol^−1^, i.e., pointing toward this process being enthalpically unfavorable as it would be expected for this likely electrostatic repulsion. Nonetheless, the Gibbs free energy indicated an exergonic process, which – given the value of −TΔ*S* at −50.5 kJ mol^−1^ – was in fact entropically driven. Previous studies on the interaction of PDMAPS with both BSA and LYZ revealed no detectable signs of binding, despite PDMAPS showing a much lower zeta potential than PIPY (Table 3) [68]. Complementary to our findings, this highlights that electrostatics do not play a central role in the (non‐)binding of PIPY and other zwitterionic polymers to proteins, thereby underlining their expected low binding affinity to biomolecules. The entropic factor of PIPY‐binding to BSA may thus originate from the displacement of relatively weak‐bound water from the protein's hydrophobic pockets in subdomains IIA and IIIA [69]. Generally, the ITC measurements showed that PIPY indeed shows minimal, surface‐charge independent binding to biomolecules that, if at all, is driven by entropic over enthalpic factors.

Based on these findings, insulin‐stability assays were conducted to assess whether PIPY can protect the protein from heat‐stress‐induced degradation. A similar approach was previously described by Herrmann et al. for an ultrahydrating dendrimeric polymer [70]. When heated, insulin forms amyloid fibrils to which the fluorescent dye Thioflavin T (ThT) can bind [71]. Upon binding, the dye's fluorescence emission peak shifts from ≈450 to 482 nm (Figure SF36, Supporting Information), making it useful as an activity assay for, for example, insulin [72]. Here, the assays were conducted for all four polymers at concentrations of 0.1, 0.5, and 1.0 mg mL^−1^ and heating the insulin solution to 50°C for 5 h in two independent experiments. All polymers showed the ability to protect insulin to differing degrees (Figure 4c). Analysis of variance (ANOVA) at p = 0.05 showed that (i) the differences between concentrations for each polymer and (ii) the differences between different polymers at the same concentration were significant. Noting this, a concentration‐dependent protective capability of PEG was observed. PEG is unique among the four polymers analyzed here as it does not show the CAC‐phenomenon below 1 mg mL^−1^. As aforementioned, this observation points toward PEG not showing intramolecular folding or changes in hydration shell at these concentrations – factors that are essential in the context of PEG being used as tool in drug formulations and protein‐conjugation [73, 74]. In contrast, a similar trend in concentration‐dependent protection was not observed for PIPY, PDMAPS and PMPC; however, is important to note that PIPY was the only polymer that prevented degradation best at a low concentration of 0.1 mg mL^−1^, compared to the optimal concentration of PMPC and PDMAPS, laying at 0.5 mg mL^−1^. In 2024, Rajan et al. proposed a mechanism for the protection of insulin from degradation using PDMAPS (Figure 4d) [18]. In short, even in the presence of excipients like PIPY, insulin unfolds with increasing temperature. Once unfolded, it would start aggregating to form amyloid fibrils. However, it was found that strongly hydrated polymer chains prevent this aggregation by forming an aggregate with the unfolded protein themselves. Due to the weak interactions, such an aggregate can not only be dissolved again, but also reform the native protein when cooled. Knowing this process occurs for PDMAPS and seeing its similarities to the other polymers, we assumed that this mechanism also holds for other hydrophilic and overall charge‐neutral polymers, including PIPY, PMPC and PEG. It is worthy noting that freely solubilized, i.e., nonaggregated, polymer chains are necessary for this process to occur. In order to assess whether higher polymer concentrations might also increase the retained insulin activity, the assays were conducted at concentrations of 5 and 10 mg mL^−1^. For all polymers, these increased concentrations accelerated the degradation of insulin upon heating, with the lowest extent observed for PEG. Based on an earlier report by Jeworrek et al., insulin seems to form amyloid fibrils more rapidly in the presence of negatively charged lipids [75]. Translating this principle to our study, we can conclude that at these high concentrations, the polymers’ net negative surface charge, as found by the prior zeta potential measurements (Table 3), supersedes the protective effect and thus accelerates insulin‐fibril formation accordingly.

All in all, PIPY showed none to only minimal entropically driven binding to biomolecules, and we could show that PIPY effectively protects insulin from heat‐induced amyloid‐fibril formation, with its optimal capabilities laying at 0.1 mg mL^−1^ compared to 0.5 mg mL^−1^ for PMPC and PDMAPS. The benefit of implementation at concentrations lower than established materials to reach similar results poses a possible advantage of PIPY and must be further explored going forth.

## 3 Conclusion

A novel chemical space for poly(iminopyridinium ylide) (PIPY) was defined in the context of nano‐ and biomedical applications. The straightforward synthesis of PIPY makes it accessible as a building block for various purposes, such as an application as protein‐stabilizing matrix, antifouling and stealth coatings and the like. We demonstrated that PIPY, as a zwitterionic and highly hydrophilic polymer, remains stable at pH values of between < 2 and 7.3, while displaying an anti‐polyelectrolyte effect in saline, underlining its applicability in physiological environments. Critical aggregation concentrations determined via fluorescence spectroscopy and DOSY NMR spectroscopy suggest that PIPY changes conformation intramolecularly below 1 mg mL^−1^, which goes along with similar properties noted for PMPC and PDMAPS. Further, our analysis revealed that PIPY rather resembles PEG upon dissolution in more apolar methanol rather than PMPC and PDMAPS. In combination with PIPY's non‐ or minimal entropically driven binding to biomolecules as well as its ability to prevent amyloid‐fibril formation upon heating of insulin at concentrations as low as 0.1 mg mL^−1^, we propose PIPY as a versatile and promising polymeric material for bio‐ and nanomedical applications. By having identified core properties of the poly(ylide) PIPY and highlighting its similarities and differences to known polymers like PEG, PMPC and PDMAPS, we were able to elucidate PIPY's promising capabilities toward replacing PEG to overcome hypersensitive reactions and as an ingredient for biomolecule‐based drug formulations. Lastly, we believe that our work will help guiding future research toward affording a fully functional biomaterial.

## 4 Experimental Section

### 4.1 Synthesis of Poly(Iminopyridinium Ylide)

#### 4.1.1 Synthesis of Monomer: Pentafluorophenyl Acrylate (PFPA)

Pentafluorophenol (1.0 eq, 10.0 g, 0.054 mol) was dissolved in dry DCM (62.5 mL) in a round‐bottom flask equipped with a magnetic stirring bar and cooled to 0°C. Then, triethylamine (TEA, 1.2 eq, 9.1 mL, 0.065 mol) was added slowly. Acryloyl chloride (1.2 eq, 5.03 mL, 0.065 mol) was then added dropwise at 0°C. The reaction mixture was allowed to warm to room temperature and stirred overnight. The resulting precipitate was filtered off and washed thoroughly with DCM. The filtrate was washed twice with aqueous HCl (pH ≈ 2), twice with saturated aqueous Na_2_CO_3_, and twice with deionized water. The organic layer was dried over anhydrous MgSO4, filtered, and concentrated under reduced pressure. The crude product was purified by column chromatography on silica gel using cyclohexane as the eluent. The product was obtained as a colorless liquid in 60% yield. ^1^H NMR (400 MHz, DMSO‐d6, δ): 6.73 (dd, J = 17.2, 0.9 Hz, 1H), 6.56 (dd, J = 17.2, 10.4 Hz, 1H), 6.38 (dd, J = 10.4, 1.0 Hz, 1H). ^13^C NMR (101 MHz, DMSO‐d6, δ): 166.90, 161.63, 140.95, 140.17, 139.33, 138.44, 136.80, 135.63, 125, 50.^19^F NMR (376 MHz, DMSO‐d6, δ): −153.54–153.83 (m, 2F), −157.76 (t, J = 23.2 Hz, 1F), −162.32–162.61 (m, 2F). FTIR (ATR, cm^−1^): 1770 (C═O ester), 1513 (C─C aromatic).

#### 4.1.2 Synthesis of Poly(Pentafluorophenyl Acrylate) (PPFPA) via RAFT Polymerization

PFPA (60 eq, 1.94 g, 8.1 mmol), DDMAT (1 eq, 0.05 g, 0.14 mmol) and AIBN (0.2 eq, 4.5 mg, 0.03 mmol) were placed into a round‐bottom flask with a magnetic stirring bar. The flask was sealed, and dry 1,4‐dioxane (4 mL) was added, and the reaction mixture was degassed with argon for 15 min. Then, the flask was immersed in a preheated oil bath of 70°C and kept there for 16 h. The reaction was cooled and exposed to air to quench the polymerization. 1,4‐Dioxane was removed under reduced pressure, and then, the reaction mixture was diluted with THF and precipitated from ice‐cold methanol to afford a white solid. The precipitation process was repeated twice. After drying under vacuum at 40°C for 24 h, 1.39 g of the poly(PFPA) was isolated (yield: 72%). ^1^H NMR (400 MHz, CDCl_3_, δ): 3.09 (br. s, 1H), 2.23 (br.m, 2H). ^19^F NMR (376 MHz, CDCl_3_, δ): −153.16 (br. s, 2F), −156.77 (br. s, 1 F), −162.19 (br. s, 2 F). FTIR (ATR, cm^−1^): 1782 (C═O ester), 1520 (C─C aromatic). SEC (THF, PS‐standard): M_n_ = 8.2 kg mol^−1^, M_w_ = 12 kg mol^−1^, Ð = 1.48

#### 4.1.3 Synthesis of 1‐Aminopyridinium Iodide (1‐API)

Hydroxylamine‐O‐sulfonic acid (1.0 eq, 3.77 g, 0.033 mol) was dissolved in distilled water (10.0 mL), and the solution was added to a 50 mL round‐bottom flask containing pyridine (3.0 eq, 8.1 mL, 0.10 mol) at room temperature. The mixture was stirred at 90°C in an oil bath for 20 min, then cooled to room temperature. Potassium carbonate (1.3 eq, 5.99 g, 0.043 mol) was added, and the volatiles were removed under reduced pressure. The resulting residue was dissolved in ethanol (25.0 mL), and the suspension was filtered under vacuum to remove insoluble residues. The filtrate was acidified to pH ≈ 5 with a 55–57 wt.% aqueous solution of hydroiodic acid (HI) and stored at −20°C overnight. A yellow precipitate formed and was collected by vacuum filtration, then washed thoroughly with diethyl ether to yield the product as a yellow solid (yield 70%). ^1^H NMR (400 MHz, DMSO‐*d6*, δ): 8.80–8.74 (m, 2H), 8.45 (s, 2H), 8.32–8.23 (m, 1H), 8.09–7.93 (m, 2H). ^13^C NMR (101 MHz, DMSO‐*d6*, δ): 140.26, 138.78, 128.70. FTIR (ATR, cm^−1^): 3180, 3050 (N—H).

#### 4.1.4 Synthesis of Poly(Iminopyridinium Ylide) (PIPY) via PPM of PPFPA

1‐API (1 eq, 0.31 g, 1.40 mmol) was dissolved in 1.5 mL of dry DMSO, and TEA (2 eq, 0.39 mL, 2.80 mmol) was added to the solution. The mixture was stirred at room temperature for 1 h. Separately, poly(PFPA) (0.9 eq, 0.3 g, 1.26 mmol) was placed into a round‐bottom flask equipped with a magnetic stirring bar with an additional 1.5 mL of dry DMSO. The previously prepared 1‐API solution was then added to the flask, and the reaction mixture was stirred for 24 h at room temperature under an Ar atmosphere in the dark. The product was isolated by precipitation into cold acetone. The precipitation process was repeated twice. After drying under vacuum at 40°C for 24 h, 0.155 g of the P(IPY) was isolated (yield: 82%). ^1^H NMR (400 MHz, DMSO‐*d6*, δ): 8.71 (br.s, 2H), 7.93 (br.s. 1H), 7.60 (br.,s. 2H), 1.85–1.69 (br. m, 2H). SEC (water, PEG‐standard): M_n_ = 26.2 kg mol^−1^, M_w_ = 36.2 kg mol^−1^, Ð = 1.38

### 4.2 Insulin Stability Assays

Hundred microliters of insulin stock (2 mg mL^−1^) in glycine buffer (pH 2.2) were added to Eppendorf tubes (1.5 mL). To each sample, 0, 10, 50, or 100 µL polymer stock (2 mg mL^−1^) in milliQ were added, corresponding to final concentrations of 0 (control), 0.1, 0.5, and 1.0 mg mL^−1^ of polymer, respectively. The samples were diluted to a final volume of 200 µL with milliQ. This procedure was carried out for PEG, PIPY, PMPC, and PDMAPS. All samples were heated at 50°C and 600 rpm. After 5 h, 10 µL of each sample was added to new Eppendorf containing 190 µL of Thioflavin T stock solution (20 µm) and centrifuged for 10 min at (check unit of centrifuge). In a quartz cuvette (check this), the samples were diluted to a final volume of 2 mL with milliQ, and their fluorescence spectrum at 450 nm excitation and 482 nm emission was measured. All spectra were background‐corrected by subtracting the spectrum of fresh, unheated insulin.

## Conflicts of Interest

The authors declare no conflicts of interest.

## Supporting information




**Supporting File**: marc70093‐sup‐0001‐SuppMat.pdf.

## Data Availability

The data that support the findings of this study are available in the supplementary material of this article.
