# Supplementary material for: Hydrophilic Poly(Iminopyridinium Ylide)s: Defining a New Chemical Space for Poly(ylide)s
Source: Macromol Rapid Commun. 2025 Oct 14;47(1):e00641. doi: 10.1002/marc.202500641 (PMC12784188; doi:10.1002/marc.202500641)
Supplement: Supplementary file 1 — Supporting File: marc70093‐sup‐0001‐SuppMat.pdf. [file MARC-47-e00641-s001.pdf]

Supporting Information for

Hydrophilic Poly(iminopyridinium ylide)s: Defining a New Chemical Space for Poly(ylide)s

Noël René Schneider,[‡,1] Aleksandra M. Orlova,[‡,2] Nuwanthika Dilrukshi Kumarage,[1]
Patrick Theato,[*,2,3] Kevin Neumann[**,1]

[1] *Institute for Molecules and Materials, Radboud University, Nijmegen, The Netherlands.*
[2] *Soft Matter Synthesis Laboratory – Institute for Biological Interfaces III (IGB-3), Karlsruhe Institute of Technology, Karlsruhe, Germany.*
[3] *Institute for Chemical Technology and Chemistry (ITCP), Karlsruhe Institute of Technology, Karlsruhe, Germany.*

‡ = contributed equally; *patrick.theato@kit.edu; **kevin.neumann@ru.nl

Contents

S1 Materials, Instrumentation and Equipment **2**
- S1.1 Materials . . . 2
- S1.2 Instrumentation and Equipment . . . 2

S2 Synthesis **3**
- S2.1 Synthesis of Poly([2-(methacryloyloxy)ethyl]dimethyl-(3-sulfopropyl)ammonium hydroxide) (PDMAPS) 3
- S2.2 Synthesis of Poly(2-methacryloyloxyethyl phosphorylcholine) (PMPC) . . . 3

S3 Physiochemical Properties and Biochemical Assays **4**
- S3.1 Stability Assays . . . 4
- S3.2 CAC Determination . . . 4
  - S3.2.1 Script for Sigmoidal Approximation of CAC . . . 4
  - S3.2.2 Script for Determination of the Hill Coefficient . . . 5
- S3.3 Zeta-Potential Measurements . . . 6
- S3.4 Diffusion-ordered spectroscopy (DOSY) NMR . . . 6
- S3.5 Isothermal Calorimetry (ITC) . . . 6

S4 Spectroscopic Results **8**
- S4.1 NMR . . . 8
  - S4.1.1 1D NMR Spectra . . . 8
  - S4.1.2 NMR Stability Assays . . . 14
  - S4.1.3 DOSY NMR . . . 16
- S4.2 FT-IR . . . 20
- S4.3 Chromatography . . . 21
- S4.4 Zeta-Potential . . . 22
- S4.5 Fluorescence Spectra of the Insulin Activity Assay . . . 23
- S4.6 Circular Dichroism of BSA and PIPY . . . 23

S1 Materials, Instrumentation and Equipment

S1.1 Materials

2,3,4,5,6-Pentafluorophenol (PFP, 99%) was purchased from BLD Pharm. 2-(dodecylthiocarbonothioylthio)-2-methylpropionic acid (DDMAT, 98%), azobisisobutyronitrile (AIBN, 98%), hydroxylamine-O-sulfonic acid (99.9%), [2-(Methacryloyloxy)ethyl]dimethyl-(3-sulfopropyl)ammonium hydroxide (DMAPS, 95%), 4,4'-Azobis(4-cyanovaleric acid) (ACVA, >98%) and 1,3,5-trioxane (≥99.0%) were purchased from Sigma-Aldrich. Acryloyl chloride (96%, stabilized with 400 ppm phenothiazine), 1,4-dioxane (99.5%, extra dry), and tetrahydrofuran (THF, 99.85%, extra dry) were obtained from Thermo Scientific Chemicals. Pyridine (99%), dichloromethane (CH_2Cl_2, 99.8%, extra dry), dimethyl sulfoxide (DMSO, 99.7%, extra dry) and aluminum oxide (Al_2O_3, Brockmann I, 40-300 µm, 60A) were purchased from Acros Organics. Hydroiodic acid (HI, 55–58%) was obtained from Fluka Chemika. Magnesium sulfate ($MgSO_4$) and potassium carbonate (K_2CO_3, ≥99%) were purchased from Carl Roth. Triethylamine (TEA, ≥99%) was obtained from Fisher Chemicals. 2-Methacryloyloxyethyl phosphorylcholine (MPC, 98%) was purchased from Fluorochem EU. MeO-PEG-OH (5 kDa) was obtained from Iris Biotech. All other solvents and reagents, including acetone, cyclohexane, methanol, and ethanol, were of analytical grade or higher and used without further purification. The ultra-pure Milli-Q water was obtained *via* a PURELAB Chorus 1 setup from Elga-Veolia. Dialysis membranes of MWCO 3500 Dalton Spectra/Por were used for purification.

S1.2 Instrumentation and Equipment

^{1}H, ^{13}C, and ^{19}F NMR spectra were recorded using a Bruker Ascend 400 NMR spectrometer operating at 400 MHz (^{1}H), 101 MHz (^{13}C), and 376 MHz (^{19}F), equipped with a BBO broadband observe probe (room-temperature) with tetramethylsilane (TMS) as the internal standard. ^{1}H NMR for the stability assays as well as diffusion-ordered spectroscopy were conducted on a Bruker-AVANCE III 500 MHz spectrometer equipped with a Prodigy BB cryoprobe at 298.15 K with the same internal standard and an acquisition frequency of 500.13 MHz. Size exclusion chromatography (SEC) measurements were carried out in THF on an Agilent Technologies 1260 Infinity II system (USA), equipped with a PLgel MIXED-E column (3 µm, 300 × 7.5 mm) and a PLgel MIXED-C column (5 µm, 300 × 7.5 mm), as well as UV and differential refractive index (RI) detectors. The operating temperature was set to 35 °C with a flow rate of 1 mL min^{-1}. The system was calibrated using polystyrene (PS) standards. Aqueous SEC measurements of PIPY were performed using an Agilent Technologies 1260 Infinity II system (USA) equipped with UV and RI detectors. The analyses were conducted in a water + 0.05% NaN_3 buffer solution using Suprema Linear-S (8 × 300 mm, 5 µm) and Suprema Linear-M (8 × 300 mm, 5 µm) columns. The operating temperature was maintained at 35 °C, and the flow rate was set to 1.0 mL min^{-1}. The system was calibrated using PEG standards. Fourier-transform infrared (FT-IR) spectra were recorded in the range of 4000–400 cm^{-1} using a Bruker Alpha II spectrometer. Zeta potentials were measured on a Zetasizer Pro from Malvern Panalyticial in a DTS1070 Disposable Folded Capillary Cell in Milli-Q. Three measurements with 10 scans of 10 seconds were averaged and analyzed using Zetasizer UltraPro ZS Explorer software. Fluorescence spectroscopy was conducted on a JASCO FP-8300 Spectrofluorometer in a reusable quartz-cuvette with a pathlength of 1 cm in both directions. 2 mL of each sample were loaded into the cuvette per measurement. The spectra were analyzed using the software Spectragryph.[1] Circular Dichroism was recorded on a JASCO J-815 CD Spectrometer in a reusable quartz-cuvette with a pathlength of 1 cm and a volume of 400 µL. Isothermal Titration Calorimetry (ITC) was conducted on a MicroCal PEAQ-ITC Automated from Malvern Panalytical and the data analyzed using MicroCal PEAQ-ITC analysis software. Heating for insulin-assays was conducted on a Eppendorf ThermoMixer C and the samples centrifuged on a Heraeus Fresco 17 Centrifuge from Thermo Scientific at 10000 1/min xg for 10 min.

S2 Synthesis

S2.1 Synthesis of PDMAPS

The RAFT polymerization of DMAPS was performed following the method reported by Gürdap et al.[2] DMAPS (197 mg, 0.71 mmol, 35 equiv.), DDMAT (7.6 mg, 0.02 mmol, 1 equiv.), and ACVA (1.8 mg, 6.56×10^{-3} mmol, 0.2 equiv.) were added to a dry Schlenk tube, followed by a degassed mixture of NaCl solution (0.5 M, 0.8 mL) and 1,4-dioxane (0.3 mL, pre-dried over Al_2O_3). The reaction mixture was stirred on ice bath for 20 min, then placed in a pre-heated oil bath at 75°C for 20 min and subsequently maintained at 70°C. Monomer conversion was monitored via ^{1}H NMR spectroscopy. After 90 min (conversion 90%), the reaction was quenched by exposure to air and cooling in an ice bath. The crude polymer was purified by dialysis (MWCO 3.5 kDa) against Milli-Q water for 24 h with one solvent exchange, followed by freeze-drying. PDMAPS was obtained as a white powder (75 mg, yield: 38%). ^{1}H NMR (400 MHz, D_2O): δ/ppm: 4.55 (br.s, $-CO_2-\mathbf{CH_2}-$), 3.85 (br.s, $-CO_2-CH_2-\mathbf{CH_2}-$), 3.65 (br.s, $-N(Me)_2-\mathbf{CH_2}-$), 3.28 (br.s, $-N\mathbf{(Me)_2}-$), 3.02 (br.s, $-\mathbf{CH_2}-SO_3^-$), 2.32 (br.s, $-\mathbf{CH_2}-CH_2-SO_3^-$), 2.06 (br.s, backbone-$CH_2$), 1.19-1.06 (br.s, backbone-Me).

S2.2 Synthesis of PMPC

PMPC synthesis was performed according to the procedure described by Beattie et al.[3] MPC (500 mg, 1.69 mmol, 50 equiv.), DDMAT (12.3 mg, 0.03 mmol, 1 equiv.), AIBN (1.1 mg, $6.8 \times 10^{\square 3}$ mmol, 0.2 equiv.), and a single crystal of 1,3,5-trioxane (internal standard) were added to a dry Schlenk tube. The mixture was dissolved in ethanol (1 mL) and deoxygenated by nitrogen bubbling on ice for 30 min. The sealed tube was then placed in a preheated oil bath at 75°C. Monomer conversion was monitored by ^{1}H NMR spectroscopy. Upon reaching 80% conversion, the reaction was quenched by exposure to air and immediate cooling in an ice bath. The resulting viscous mixture was redissolved with minimal brine and precipitated into an excess of acetone/methanol (17:1, v/v). The crude polymer was purified by dialysis (MWCO 3.5 kDa) against Milli-Q water for 24 h with one solvent exchange, followed by freeze-drying. PMPC was obtained as a white powder (262 mg, yield: 66%). ^{1}H NMR (400 MHz, D_2O): δ/ppm: 4.34 (br.s, $-CO_2-\mathbf{CH_2}-$), 4.26 (br.s, $-PO_4^--\mathbf{CH_2}-$), 4.13 (br.s, $-CO_2-CH_2-\mathbf{CH_2}-$), 3.73 (br.t, $-PO_4^--CH_2-\mathbf{CH_2}-$), 3.28 (s, $-N-\mathbf{Me_3}$), 1.97 (br.s, backbone-Me), 0.97 (s, backbone-CH_2), 1.94 (s, backbone-CH_2).

S3 Physiochemical Properties and Biochemical Assays

S3.1 Stability Assays

Two distinct PBS buffers in D_2O were prepared and adapted to pH = 5.7 and pH = 7.3 using DCl and NaOD. PIPY (ca. 5 mg) was dissolved in 0.5 mL of each of the buffers. The ^{1}H NMR spectra were measured every 30 minutes over the course of 24 h.

S3.2 CAC Determination

Stock solutions of each polymer (2 mg mL^{-1}) and rhodamine 6G (250 µM) were prepared in Milli-Q water. Aliquots of rhodamine 6G stock solution (20 µM) were added to appropriate volumes of polymer stock solutions and diluted with Milli-Q water or saline (1M) to a final volume of 2 mL, yielding polymer concentrations of 0.05, 0.1, 0.25, 0.5, 0.75, 1.0, and 1.2 mg mL^{-1}. Fluorescence emission was recorded at 552 nm upon excitation at 530 nm. Data points were fitted to a sigmoidal model using a Python script (subsubsection S3.2.1), and the critical aggregation concentration (CAC) was determined according to Equation 1. The optimized parameters L and CAC were then used in a second script (subsubsection S3.2.2) to calculate the Hill coefficients according to Equation 2.

$$y = \frac{L}{1 + e^{(-k \cdot (x - CAC))}} + b \tag{1}$$

where x = concentration [mg mL^{-1}]; L = maximal fluorescence intensity [a.u.]; k = slope [mL mg^{-1}]; b = y-intercept [a.u.]

$$y = \frac{L \cdot x^n}{CAC^n \cdot x^n} \tag{2}$$

where x = concentration [mg mL^{-1}]; L = maximal fluorescence intensity [a.u.]; n = Hill coefficient

S3.2.1 Script for Sigmoidal Approximation of CAC

```
import numpy as np
import matplotlib.pyplot as plt
from scipy.optimize import curve_fit

# Measurement Points
x = np.array([X1, X2, X3, ...])
y = np.array([Y1, Y2, Y3, ...])

# Sigmoidal Function
def sigmoid(x, L, x0, k, b):
    return L / (1 + np.exp(-k*(x-x0))) + b

# Guessing Initial Parameters Based on Data Points
initial_guess = [max(y), np.median(x), 1, min(y)]

# Curve Fitting
params, _ = curve_fit(sigmoid, x, y, p0=initial_guess)

# Plot Curve
x_fit = np.linspace(min(x), max(x), 100)
y_fit = sigmoid(x_fit, *params)

# Make Figure of Curve
plt.scatter(x, y, label="Data Points", color="blue")
plt.plot(x_fit, y_fit, label="Sigmoidal Fit", color="red")
plt.xlabel("x")
plt.ylabel("y")
plt.title("Sigmoidal Curve Fit")
plt.legend()
plt.show()
```

```
# Print Fitting Parameters
print(f"Fitted parameters: L={params[0]}, x0={params[1]}, k={params[2]}, b={params[3]}")
```

S3.2.2 Script for Determination of the Hill Coefficient

```
import numpy as np
import matplotlib.pyplot as plt
from scipy.optimize import curve_fit

# Measurement Points
x = np.array([X1, X2, X3, ...])
y = np.array([Y1, Y2, Y3, ...])

# Sigmoidal Function
def sigmoid(x, L, x0, k, b):
    return L / (1 + np.exp(-k * (x - x0))) + b

# Fit to Sigmoidal Model
sigmoid_guess = [max(y), np.median(x), 1, min(y)]
sigmoid_params, _ = curve_fit(sigmoid, x, y, p0=sigmoid_guess)
CAC_fixed = sigmoid_params[1]  # Extract fitted CAC

# Fixed Parameter
ymax_fixed = max(y)  # Use the max fluorescence intensity for consistency

# Hill equation (fitting only n)
def hill_equation_fixed_K_ymax(x, n):
    return (ymax_fixed * x**n) / (CAC_fixed**n + x**n)  # Fixed ymax and CAC

# Initial guess for Hill coefficient
n_guess = [2]  # Only fitting n

# Fit Hill model (only optimizing n)
hill_params, _ = curve_fit(hill_equation_fixed_K_ymax, x, y, p0=n_guess)

# Generate fitted curves
x_fit = np.linspace(min(x), max(x), 100)
sigmoid_fit = sigmoid(x_fit, *sigmoid_params)
hill_fit_fixed = hill_equation_fixed_K_ymax(x_fit, *hill_params)

# Plot results
plt.scatter(x, y, label="Data Points", color="blue")
plt.plot(x_fit, sigmoid_fit, label="Sigmoidal Fit", linestyle='dashed', color="red")
plt.plot(x_fit, hill_fit_fixed, label="Hill Fit (Fixed CAC & ymax)", linestyle='solid', color="green")
plt.xlabel("Concentration")
plt.ylabel("Fluorescence Intensity")
plt.title("Comparison: Sigmoidal vs. Hill Model (Fitting Only n)")
plt.legend()
plt.show()

# Display fitted parameters
print(f"Sigmoidal Fit Parameters: L={sigmoid_params[0]}, CAC (x0)={sigmoid_params[1]},
k={sigmoid_params[2]}, b={sigmoid_params[3]}")
print(f"Hill Fit Parameter (Fitting Only n): Hill coefficient (n)={hill_params[0]}")
```

S3.3 Zeta-Potential Measurements

Polymer solutions (c = 2 mg mL^{-1}) were prepared in Milli-Q water (≈500 µL each). Zeta potentials were measured in triplicate for each sample and averaged.

S3.4 DOSY NMR

^1H NMR spectra were recorded on a Bruker-AVANCE III 500 MHz spectrometer equipped with a Prodigy BB cryoprobe at 298.15 K, with a ^1H frequency of 500.13 MHz. Two-dimensional (2D) DOSY spectra were acquired using double stimulated echo experiment with 3 spoil gradients for convection compensation (dstebpgp3s). All spectra were acquired using 16 gradient levels, ranging from a low gradient strength of 2% to a high gradient strength of 95% with the below parameters in both solvents. Each spectrum was manually baseline- and phase-corrected and the DOSY maps were generated using the automatic processing option in MestReNova.

Table 1: Experimental parameters for the DOSY NMR experiments.

Parameter	Abbreviation	Magnitude
Duration of Gradient Pulse	p30	1 ms
Relaxation Delay	d1	1-3 s
Diffusion Time Parameter	d20	150-300 ms

S3.5 Isothermal Calorimetry (ITC)

Stock solutions of bovine serum albumin (BSA, 1 mM), lysozyme (LYZ, 1 mM), and PIPY (0.1 mM) were prepared in PBS buffer (pH 7.4). Protein solutions (syringe) were titrated into the polymer solution (cell), and the associated binding heats were recorded. Reference titrations included: buffer into polymer, protein into buffer, and buffer into buffer.

PIPY Shows Weak-Entropic Binding to **BSA**

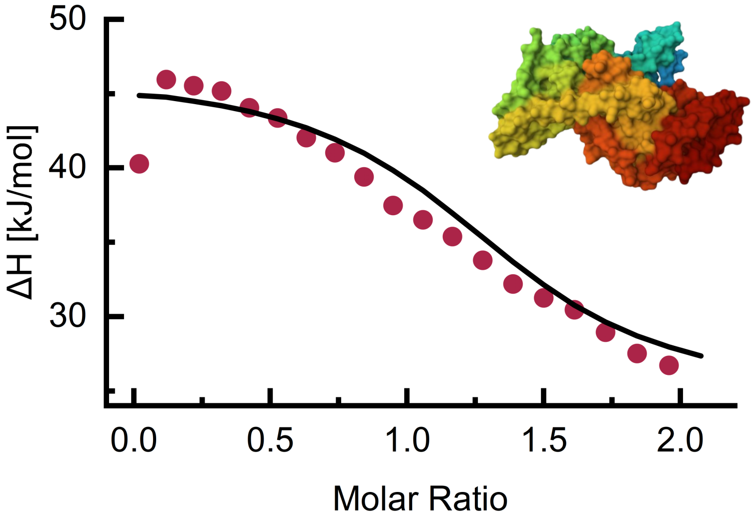

N (sites)	1.34 ± 0.135
K_D [M]	$8.82*10^{-6} \pm 5.31*10^{-6}$
ΔH [kJ/mol]	21.6 ± 3.98
ΔG [kJ/mol]	-28.9
-TΔS [kJ/mol]	-50.5

Figure SF1: isothermal calorimetry (ITC) results of mixing PIPY with bovine serum albumin (BSA).

PIPY Shows No Binding to **Lysozyme**

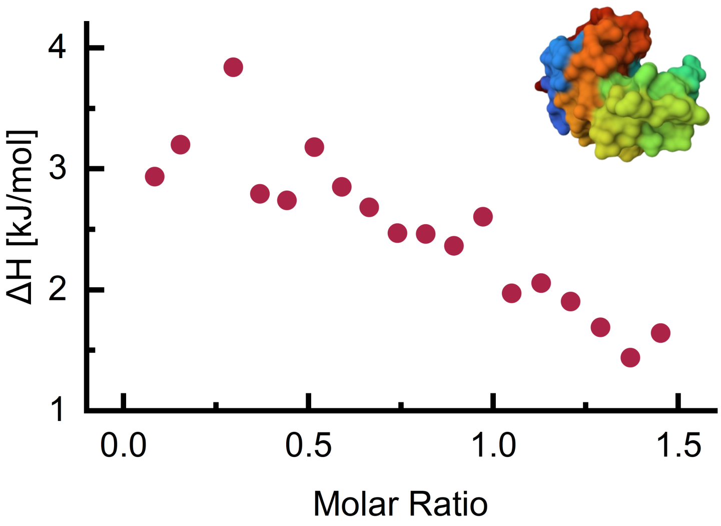

N (sites)	N/A
K_D [M]	N/A
ΔH [kJ/mol]	N/A
ΔG [kJ/mol]	N/A
-TΔS [kJ/mol]	N/A

Figure SF2: isothermal calorimetry (ITC) results of mixing PIPY with lysozyme (LYZ).

S4 Spectroscopic Results

S4.1 NMR

S4.1.1 1D NMR Spectra

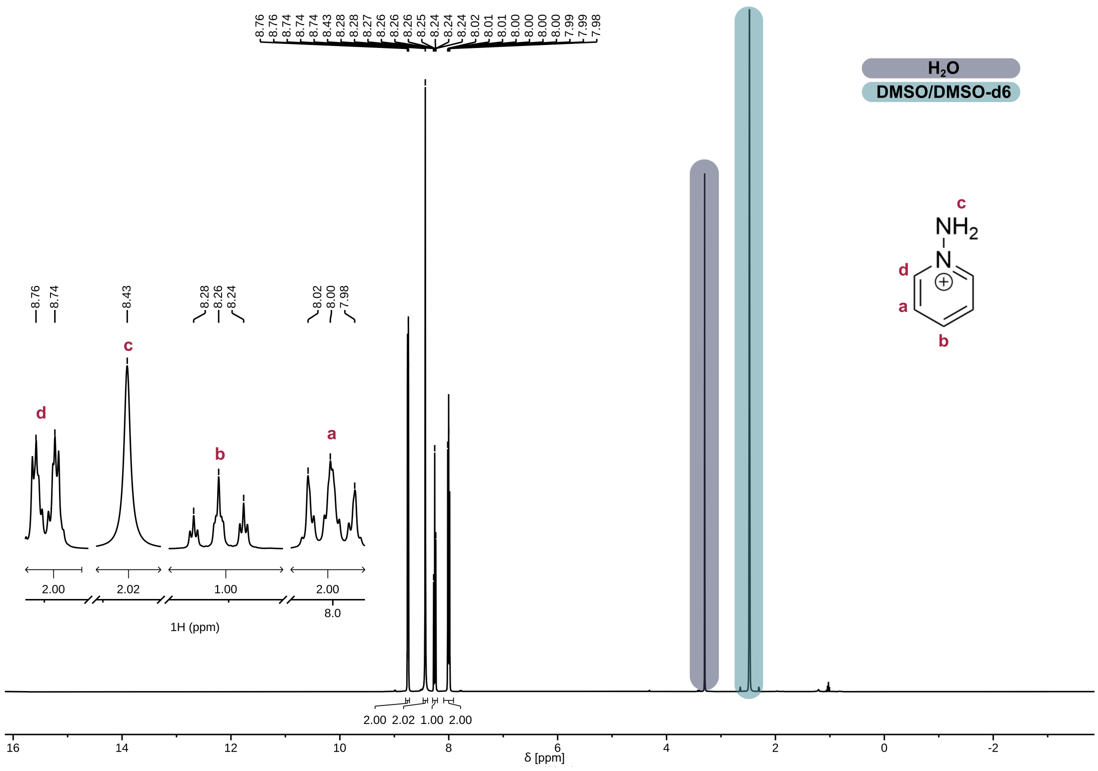

Figure SF3: ^{1}H-NMR (400 MHz, DMSO-d6, 298 K) of 1-aminopyridinium idodide (1-API).

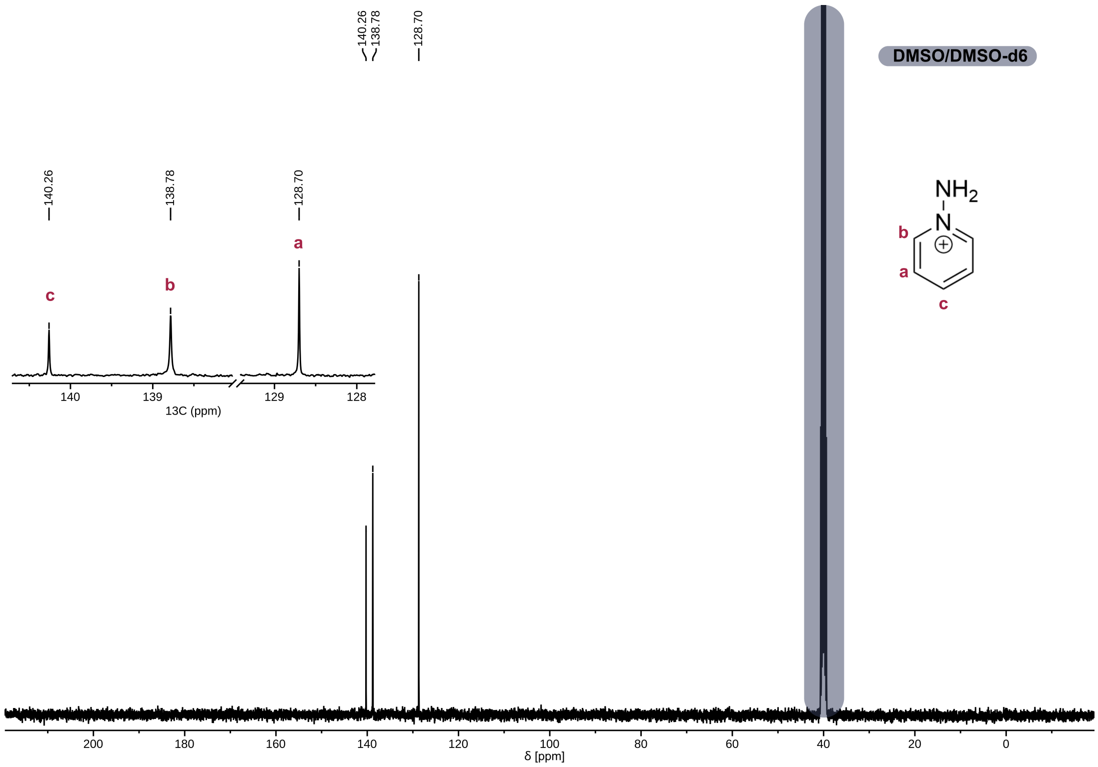

Figure SF4: ^{13}C{^{1}H} NMR (101 MHz, DMSO-d6, 298 K) of 1-aminopyridinium idodide (1-API).

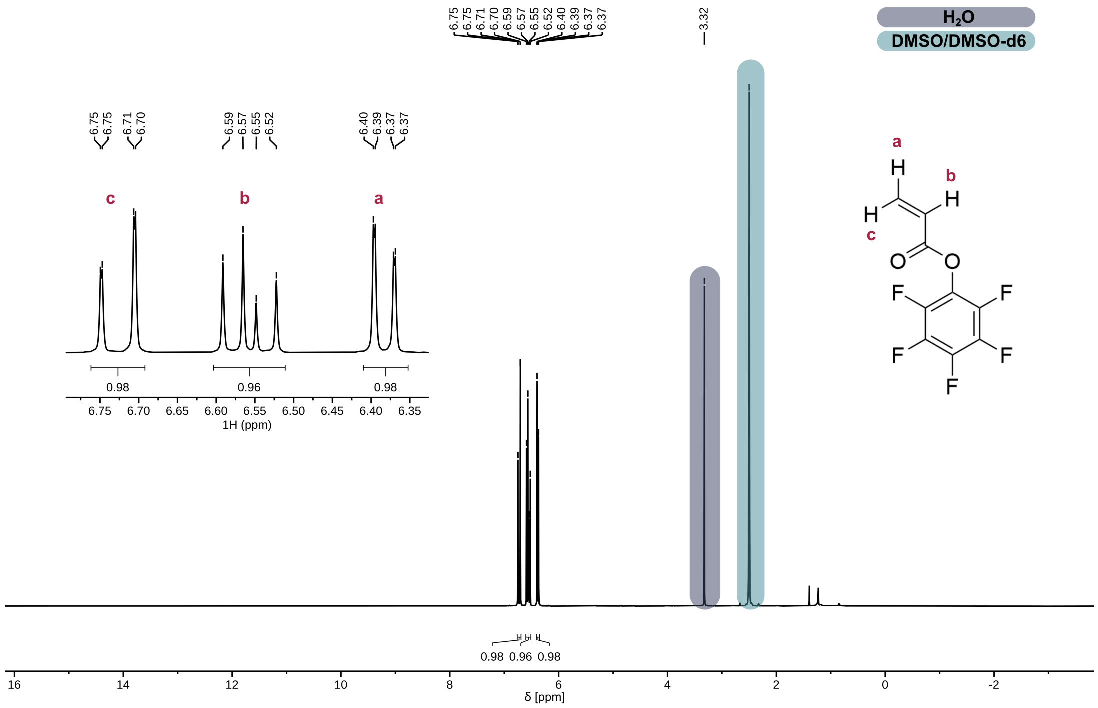

Figure SF5: ^{1}H-NMR (400 MHz, DMSO-d6, 298 K) of pentafluorophenyl acrylate (PFPA).

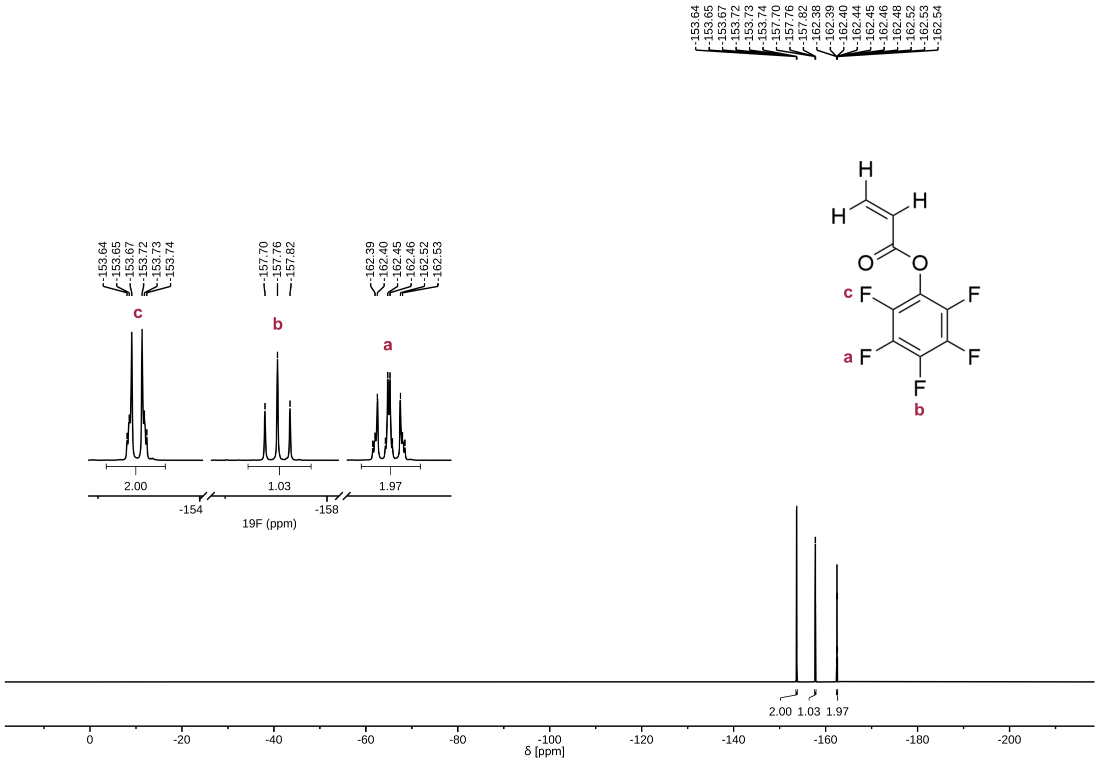

Figure SF6: ^{19}F{^{1}H} NMR (376 MHz, DMSO-d6, 298 K) of pentafluorophenyl acrylate (PFPA).

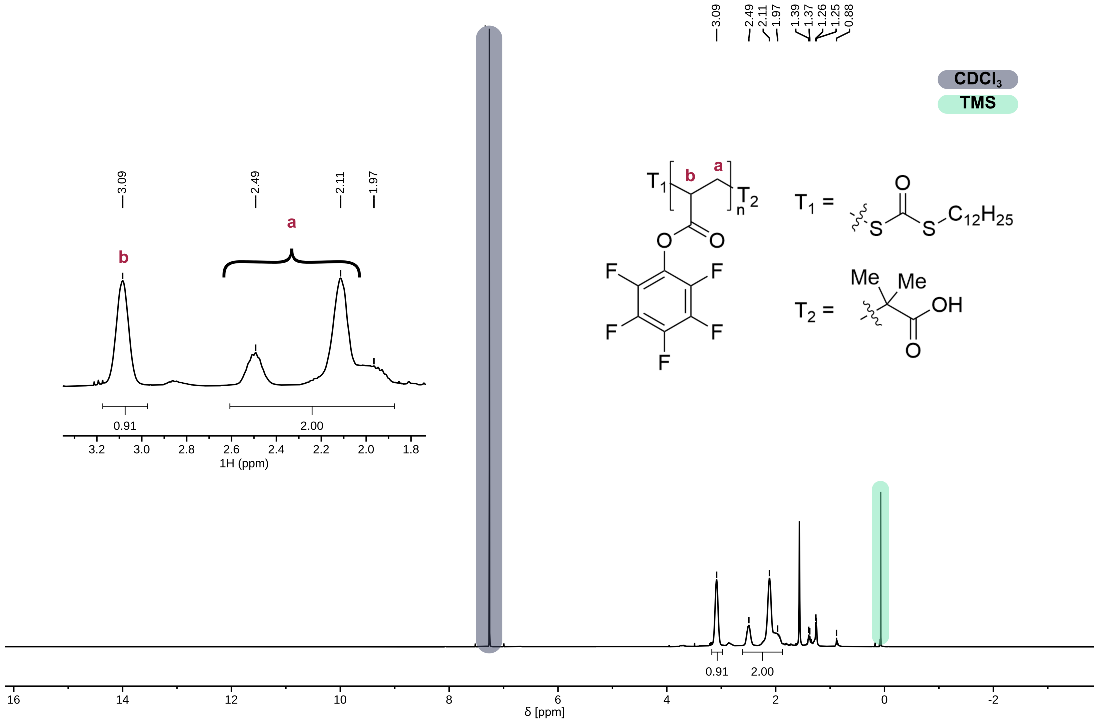

Figure SF7: ^{1}H-NMR (400 MHz, $CDCl_3$, 298 K) of poly(pentafluorophenyl acrylate) (PPFPA).

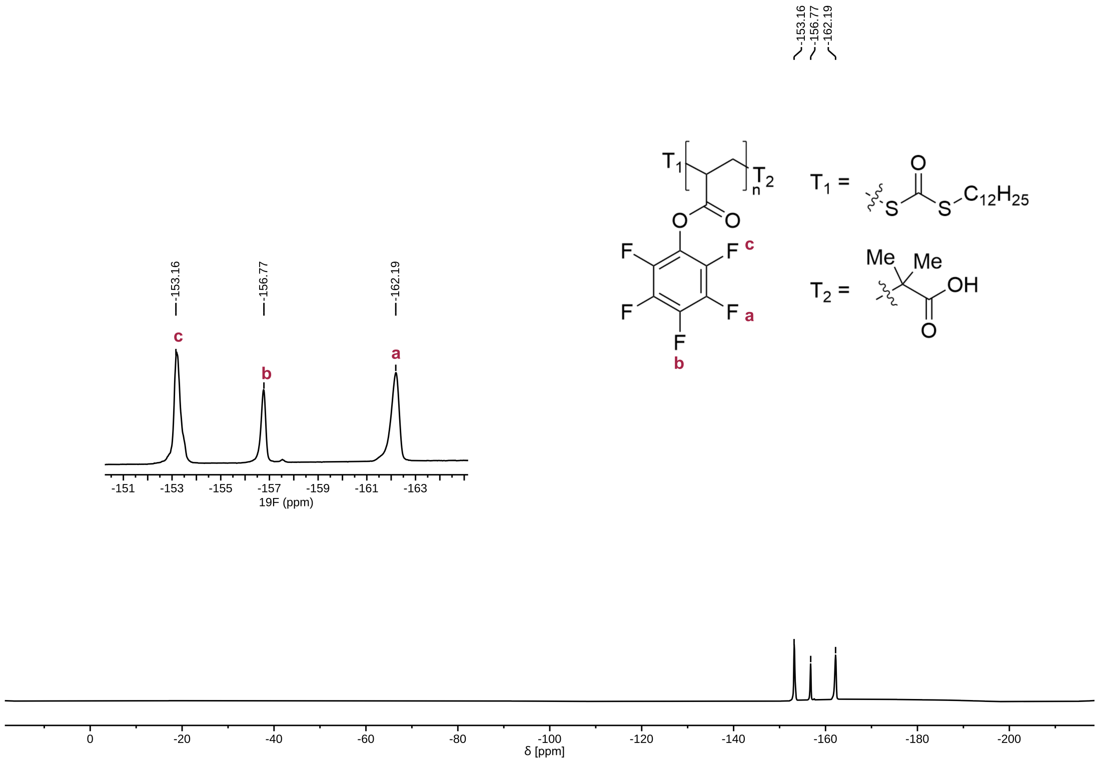

Figure SF8: ^{19}F{^{1}H} NMR (376 MHz, DMSO-d6, 298 K) of poly(pentafluorophenyl acrylate) (PPFPA) in $CDCl_3$.

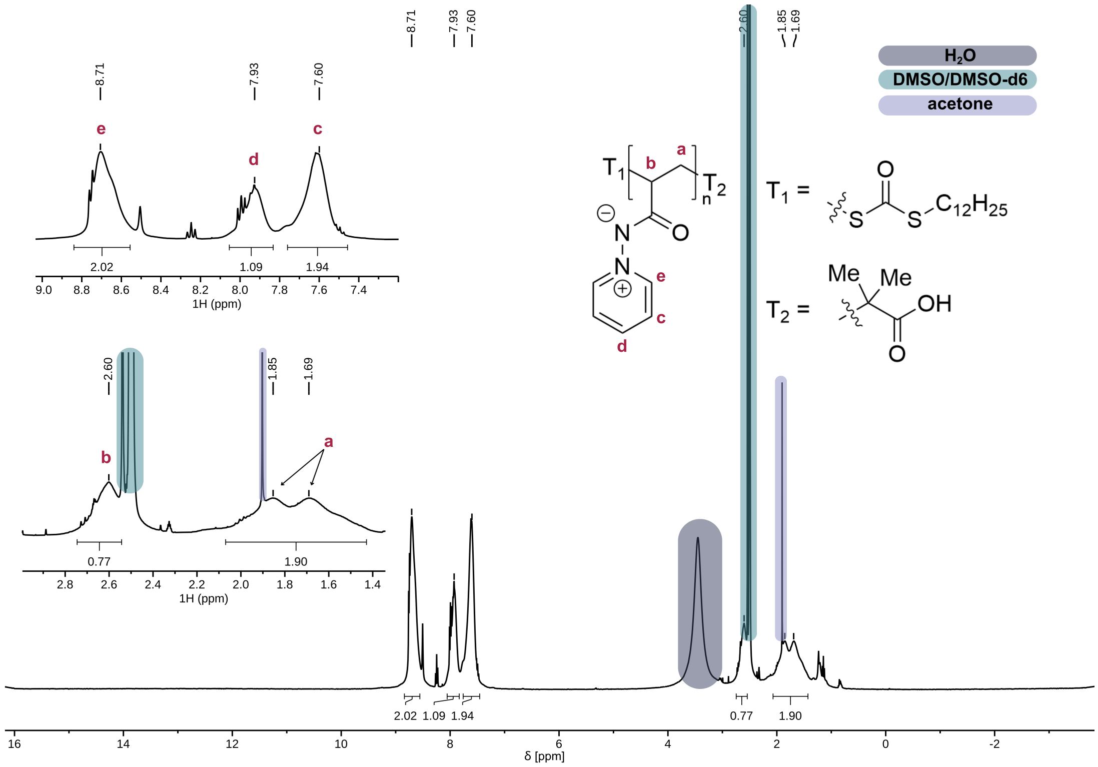

Figure SF9: ^{1}H-NMR (400 MHz, DMSO-d6, 298 K) of poly(iminopyridinium ylide) (PIPY).

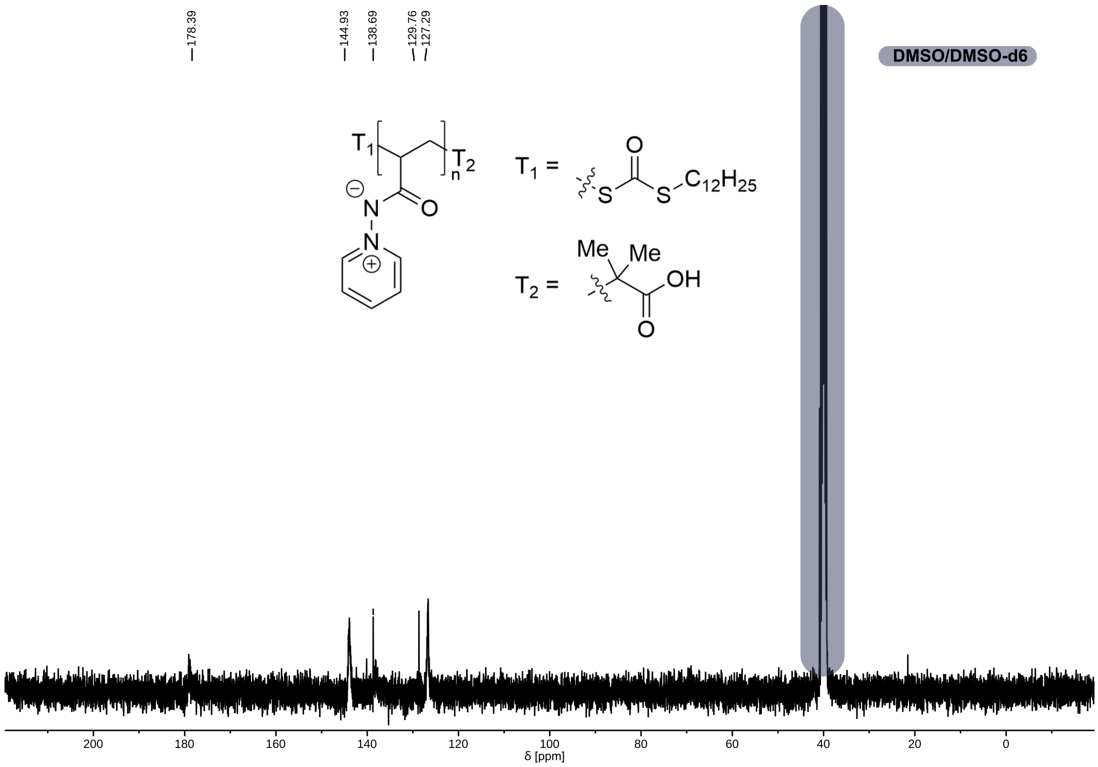

Figure SF10: ^{13}C{^{1}H} NMR (101 MHz, DMSO-d6, 298 K) spectrum of poly(iminopyridinium ylide) (PIPY).

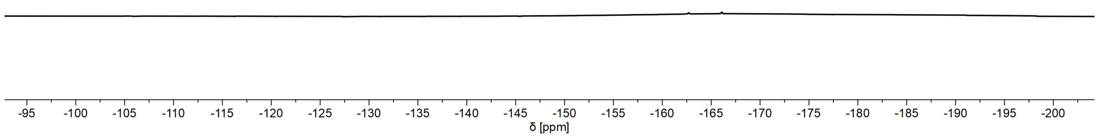

Figure SF11: $^{19}F\{^{1}H\}$ NMR (376 MHz, DMSO-d6, 298 K) of poly(iminopyridinium ylide) (PIPY) in DMSO-d6.

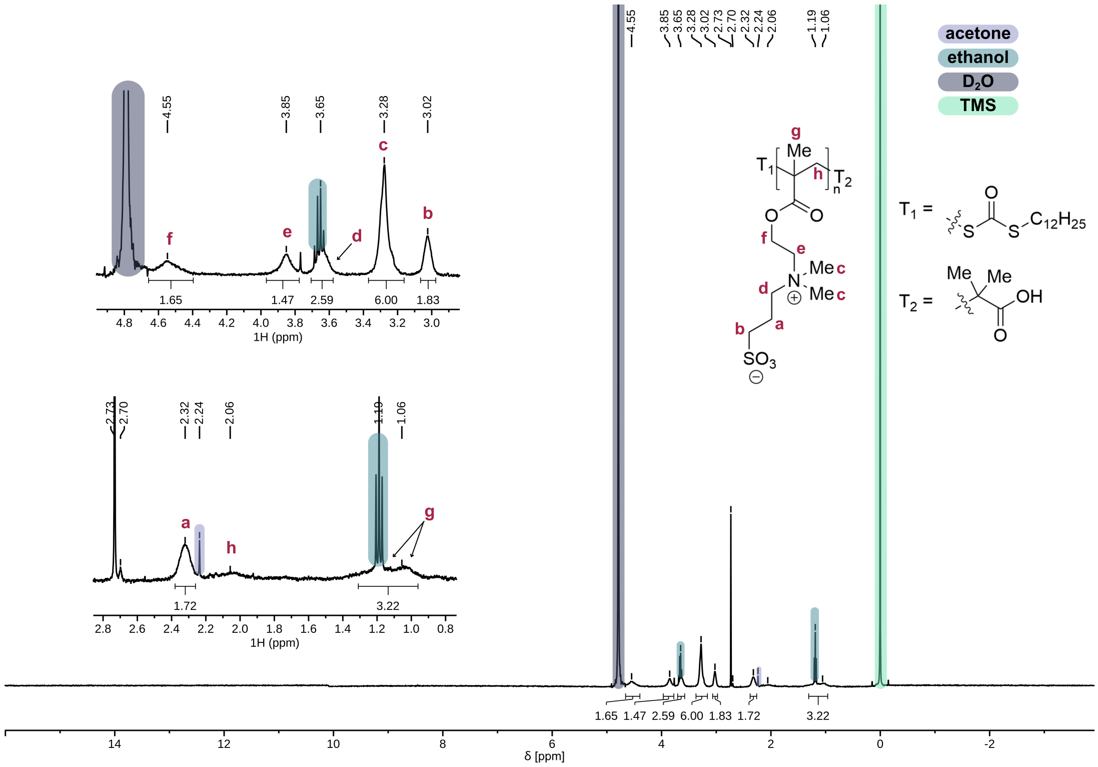

Figure SF12: ^{1}H NMR (400 MHz, D_2O, 298 K) of poly([2-(methacryloyloxy)ethyl]dimethyl-(3-sulfopropyl)ammonium hydroxide) (PDMAPS).

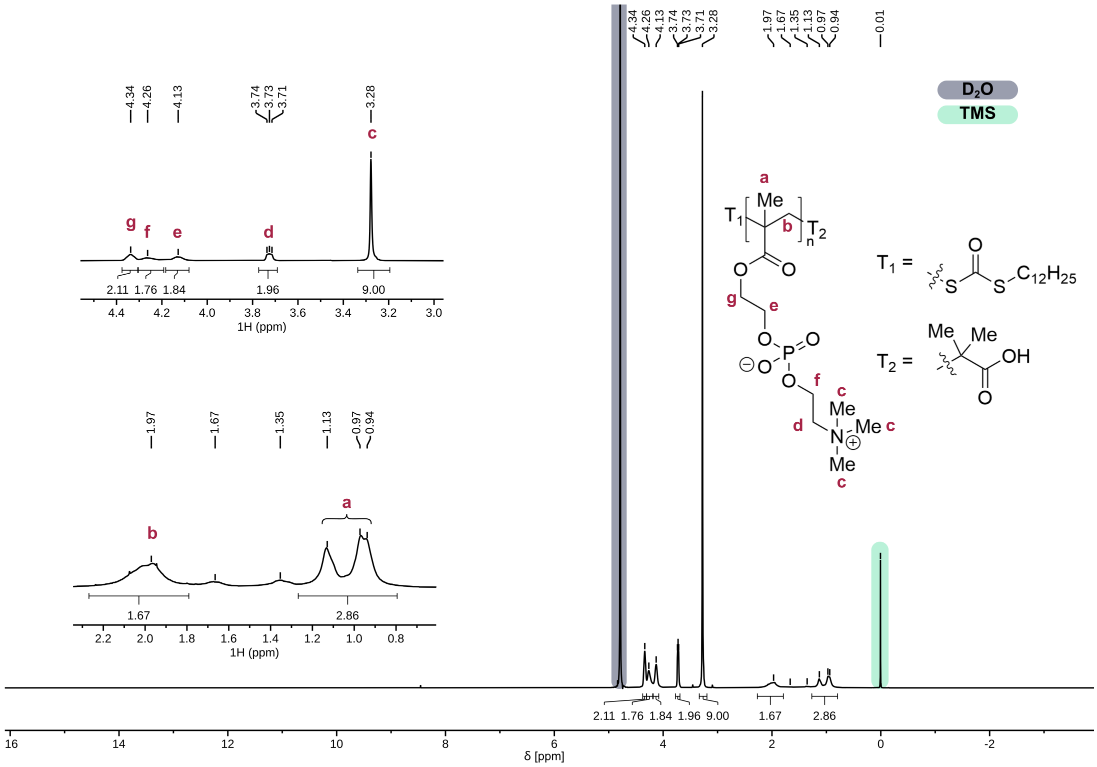

Figure SF13: ^{1}H NMR (400 MHz, D_2O, 298 K) of poly(2-methacryloyloxyethyl phosphorylcholine) (PMPC).

S4.1.2 NMR Stability Assays

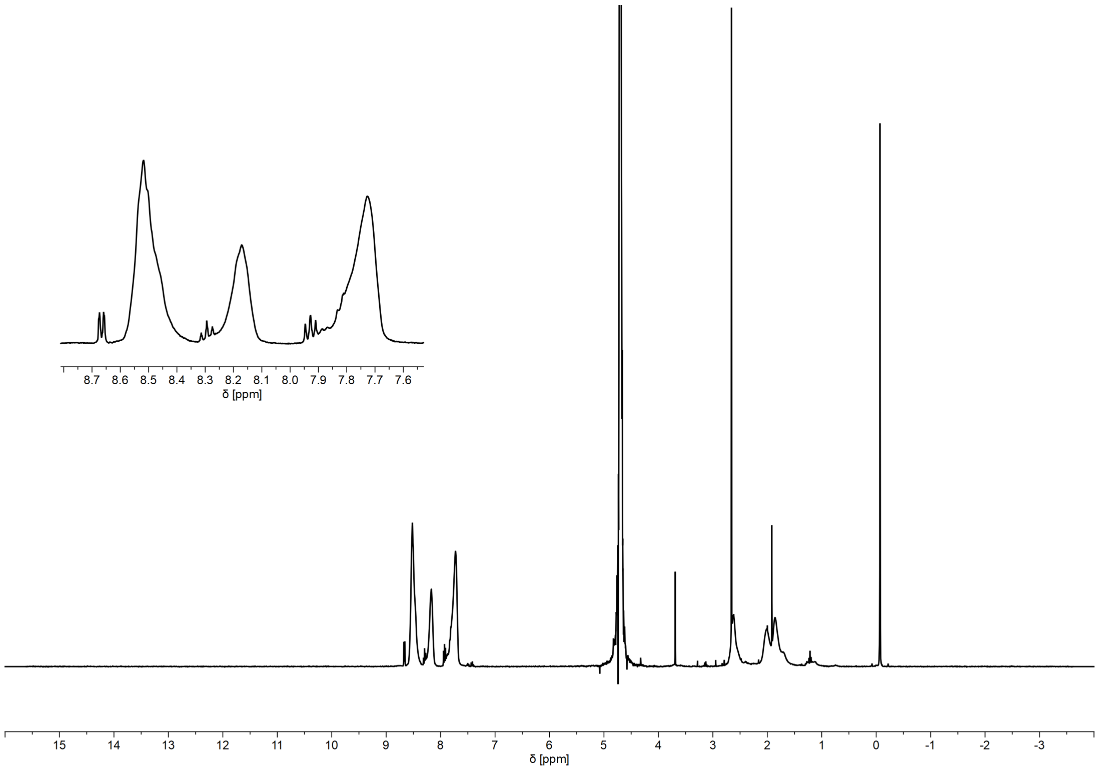

Figure SF14: ^{1}H NMR of poly(iminopyridinium ylide) (PIPY) in D_2O at pH = 5.7 after 34 days.

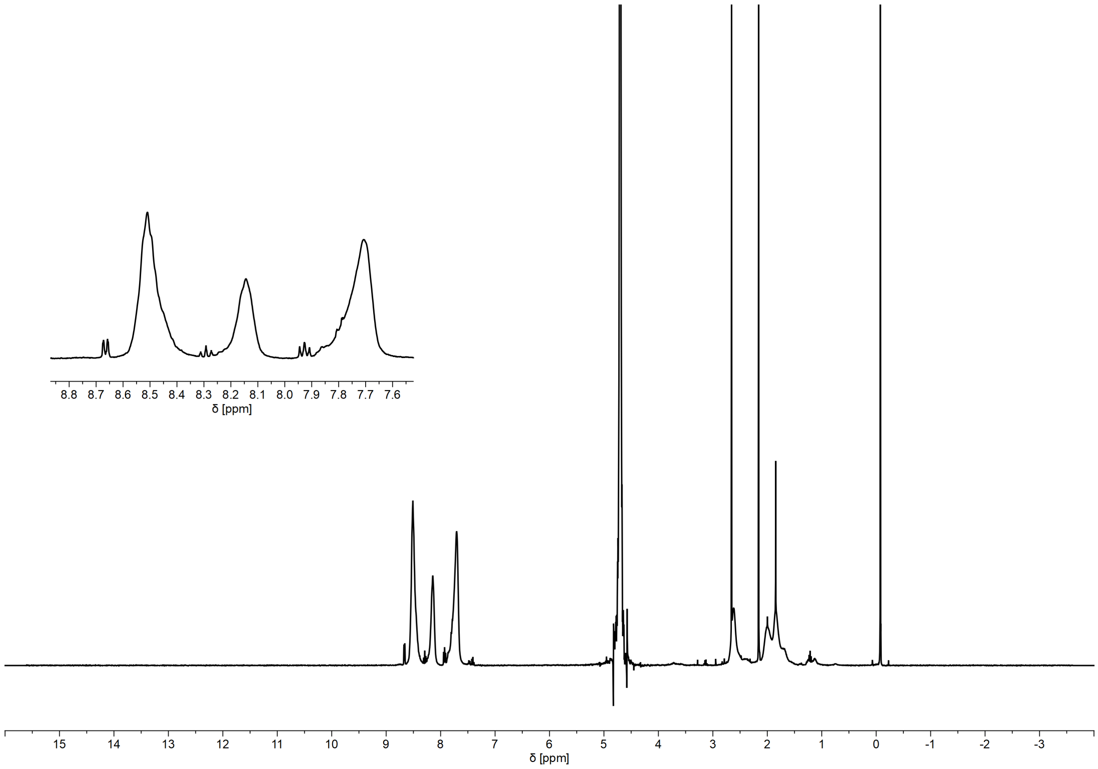

Figure SF15: ^{1}H NMR of poly(iminopyridinium ylide) (PIPY) in D_2O at pH = 7.3 after 34 days.

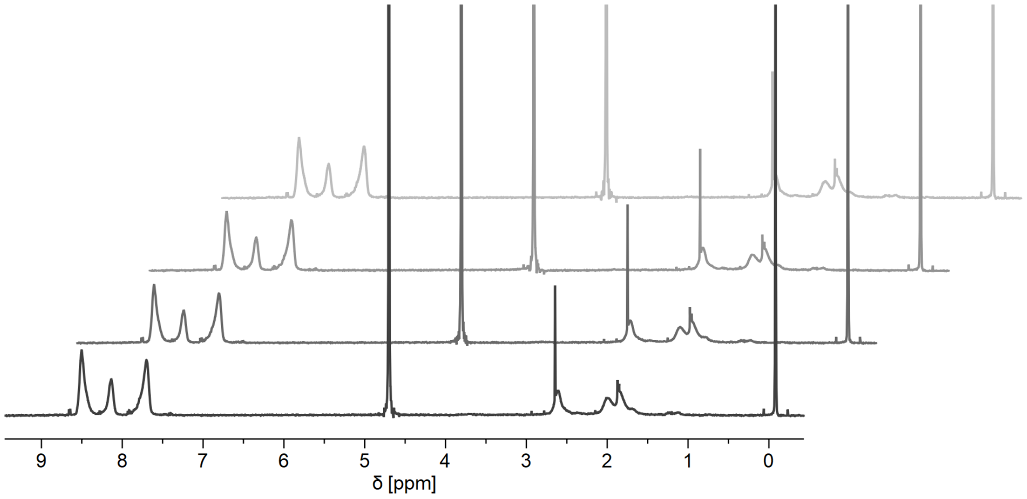

Figure SF16: ^{1}H NMR of poly(iminopyridinium ylide) (PIPY) in D_2O under daylight-irradiation at (from front to back) t = 0 h, 2 h, 3 h and 7 h.

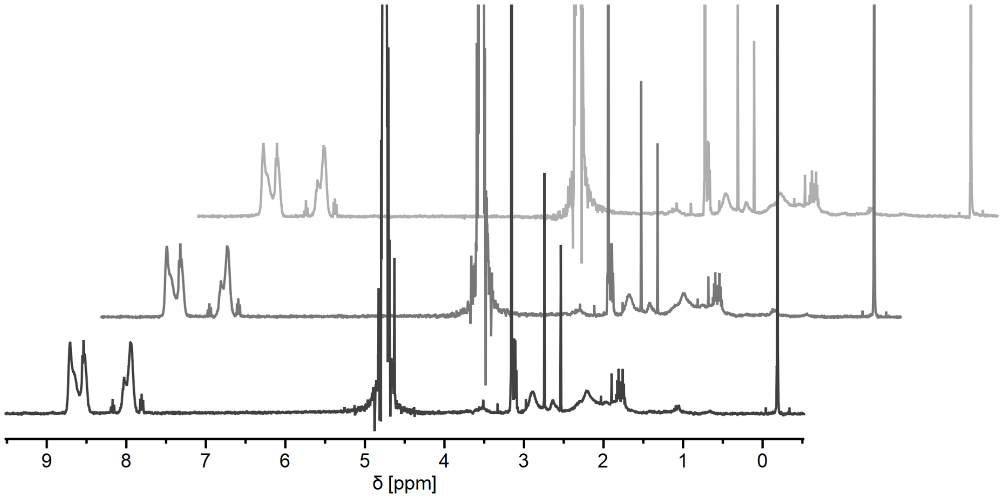

Figure SF17: ^{1}H NMR of poly(iminopyridinium ylide) (PIPY) in D_2O with DCl (pH < 2.0) at (from front to back) t = 0 h, 2 h, 48 h.

S4.1.3 DOSY NMR

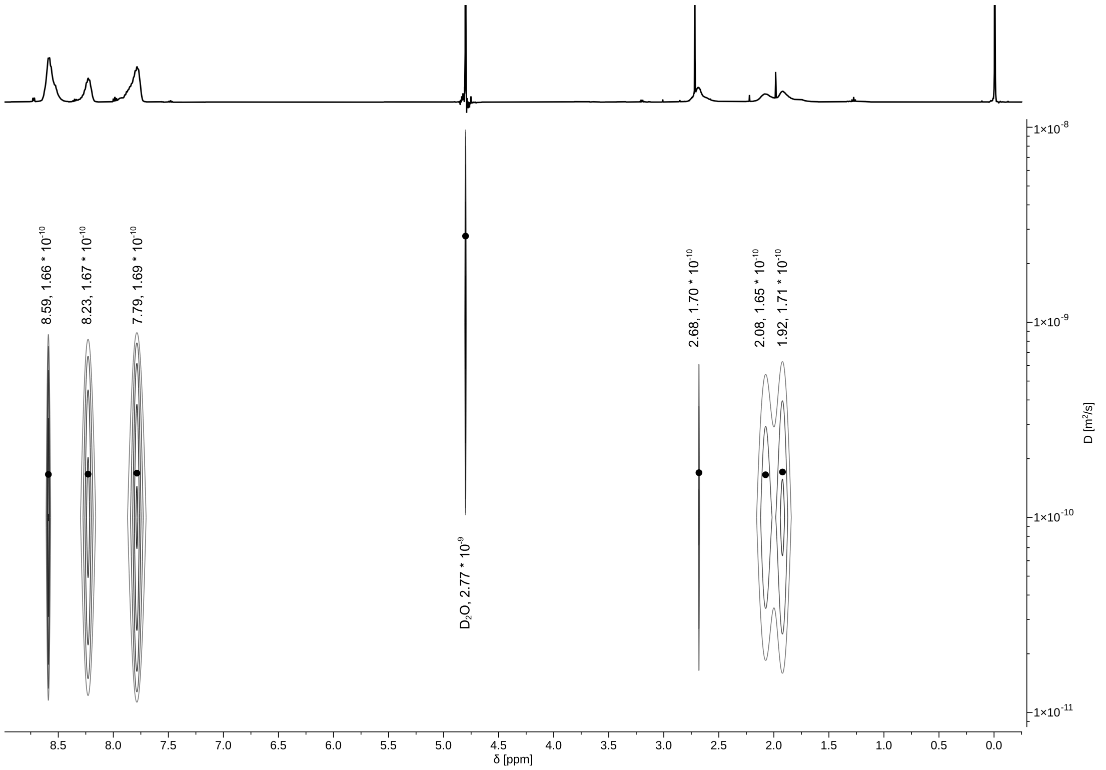

Figure SF18: DOSY NMR of poly(iminopyridinium ylide) (PIPY) in D_2O.

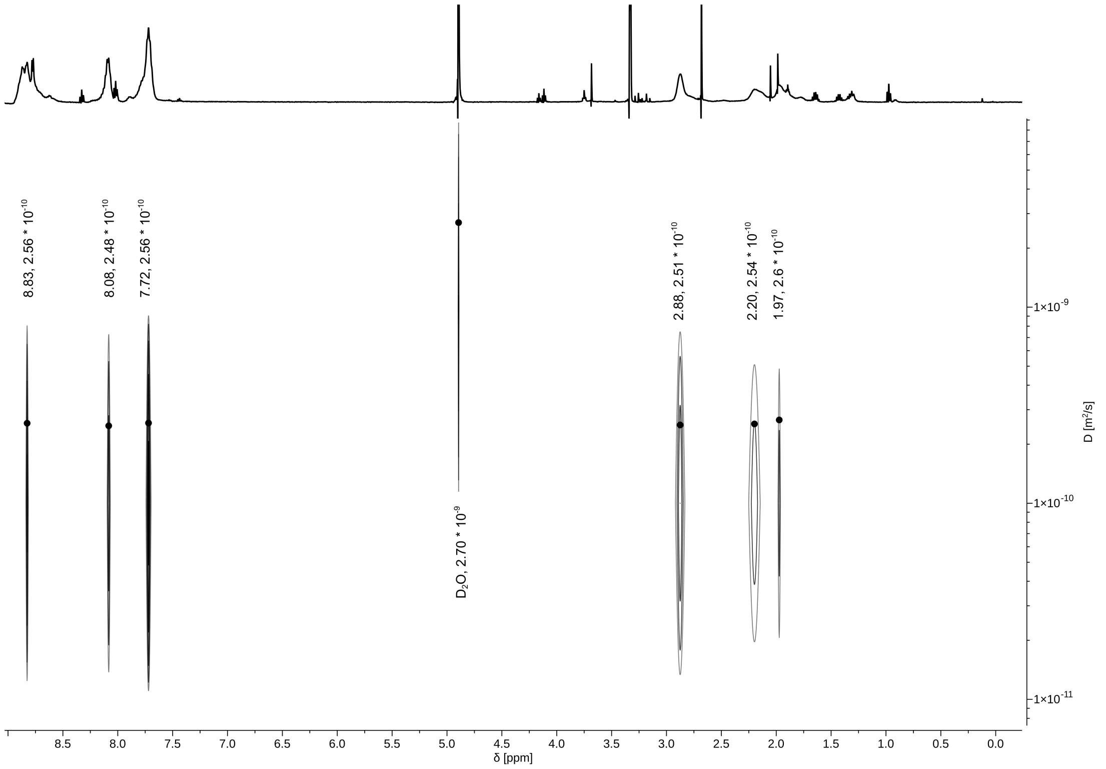

Figure SF19: DOSY NMR of poly(iminopyridinium ylide) (PIPY) in MeOD.

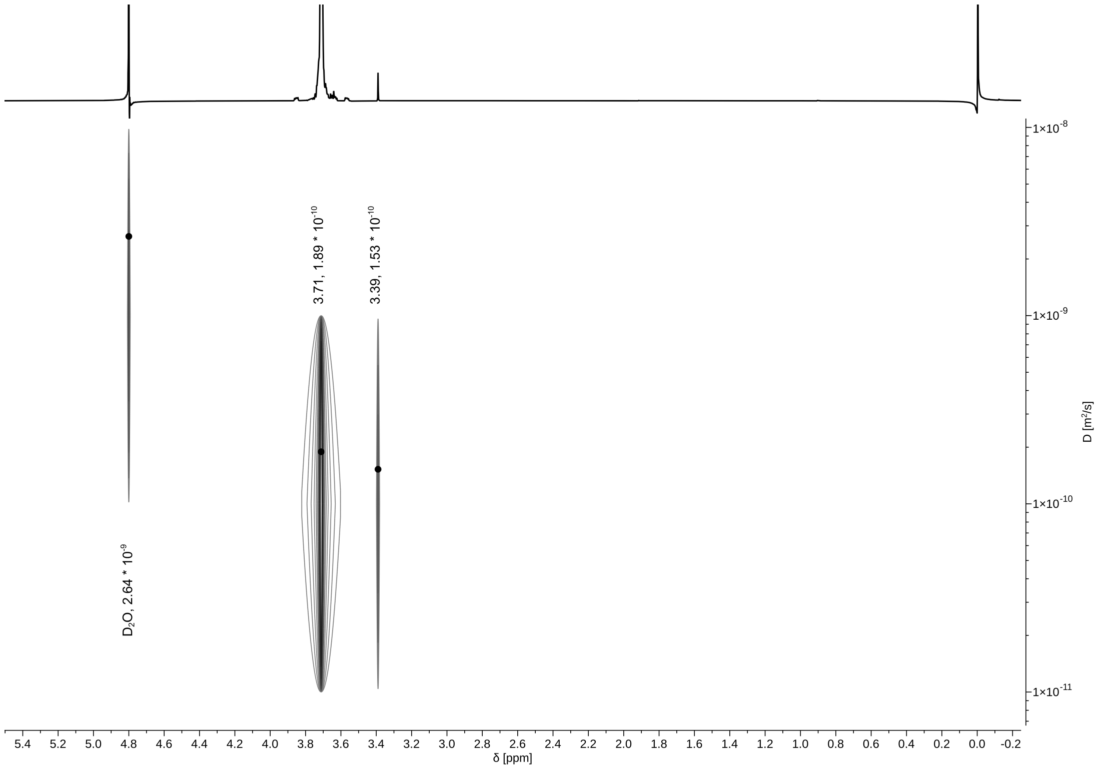

Figure SF20: DOSY NMR of poly(ethylene glycol) (PEG) in D_2O.

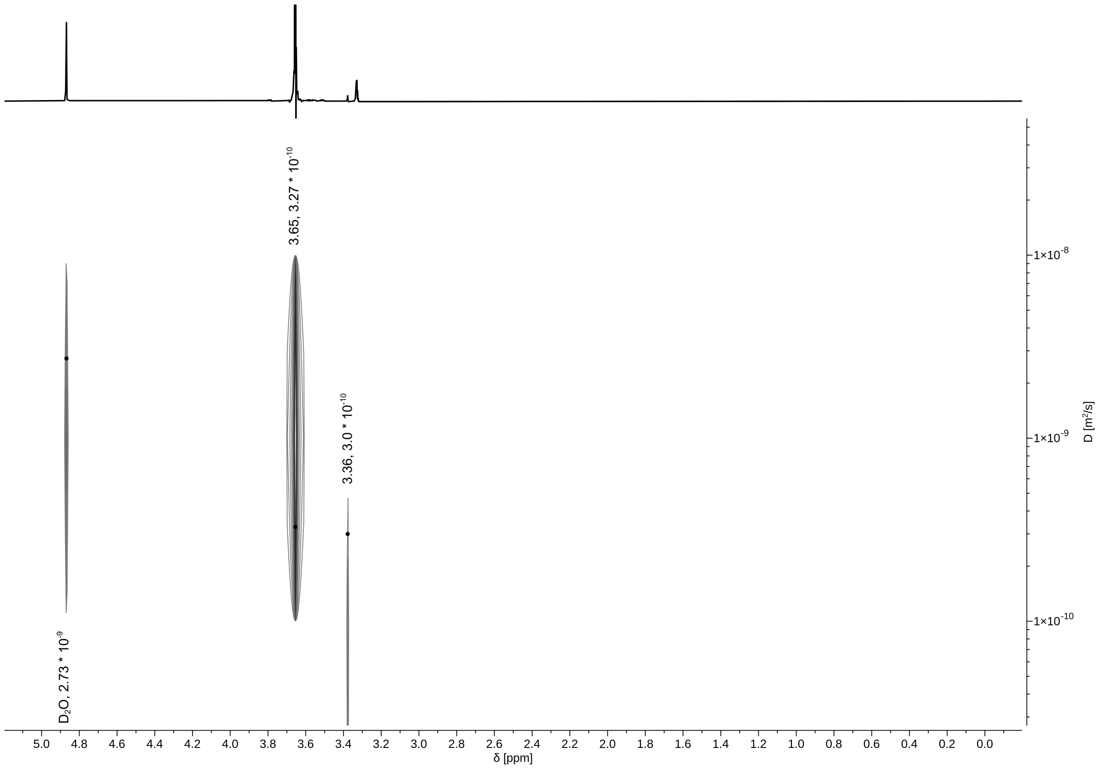

Figure SF21: DOSY NMR of poly(ethylene glycol) (PEG) in MeOD.

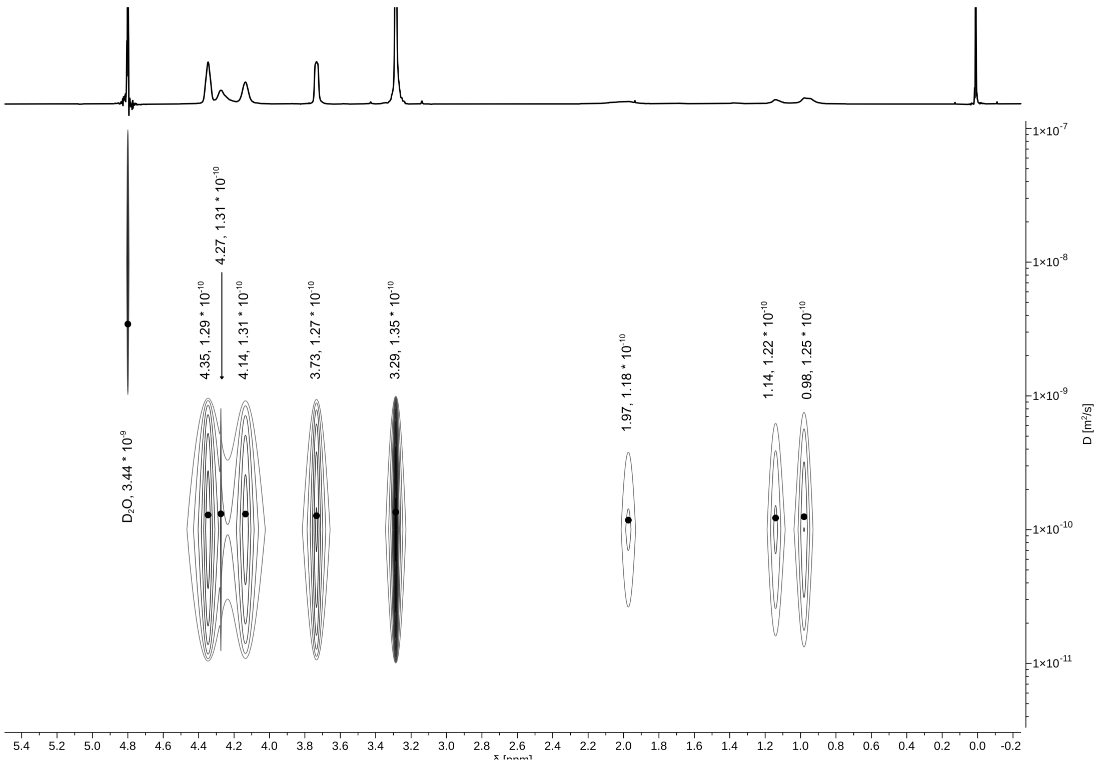

Figure SF22: DOSY NMR of poly(2-methacryloyloxyethyl phosphorylcholine) (PMPC) in D_2O.

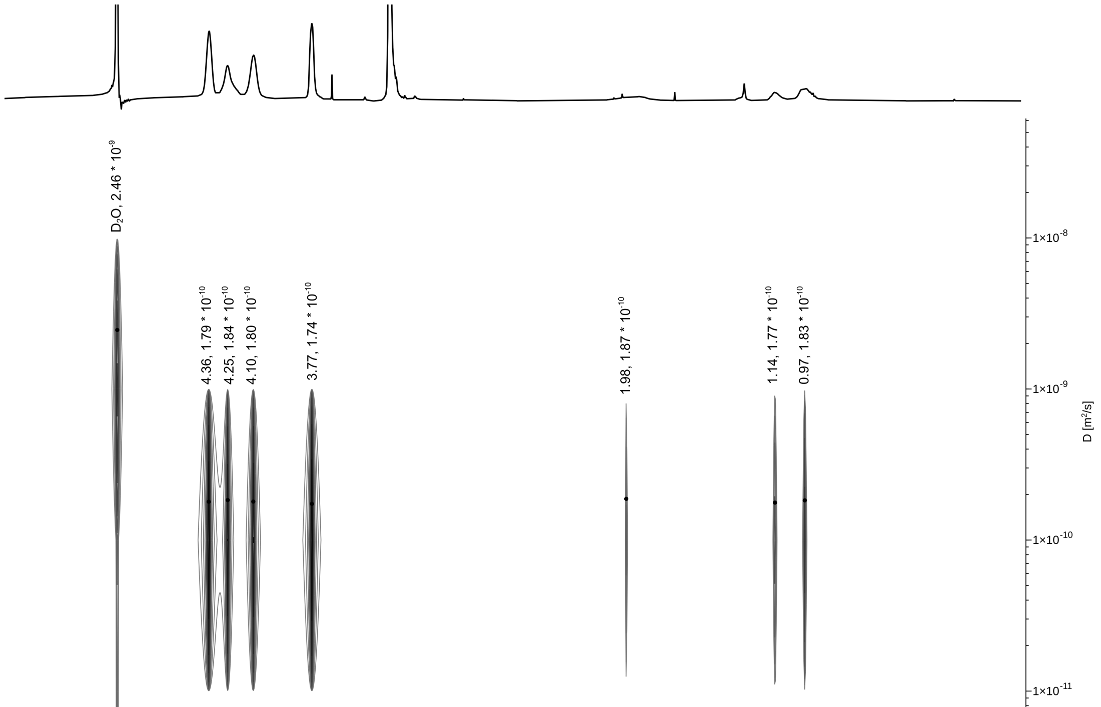

Figure SF23: DOSY NMR of poly(2-methacryloyloxyethyl phosphorylcholine) (PMPC) in MeOD.

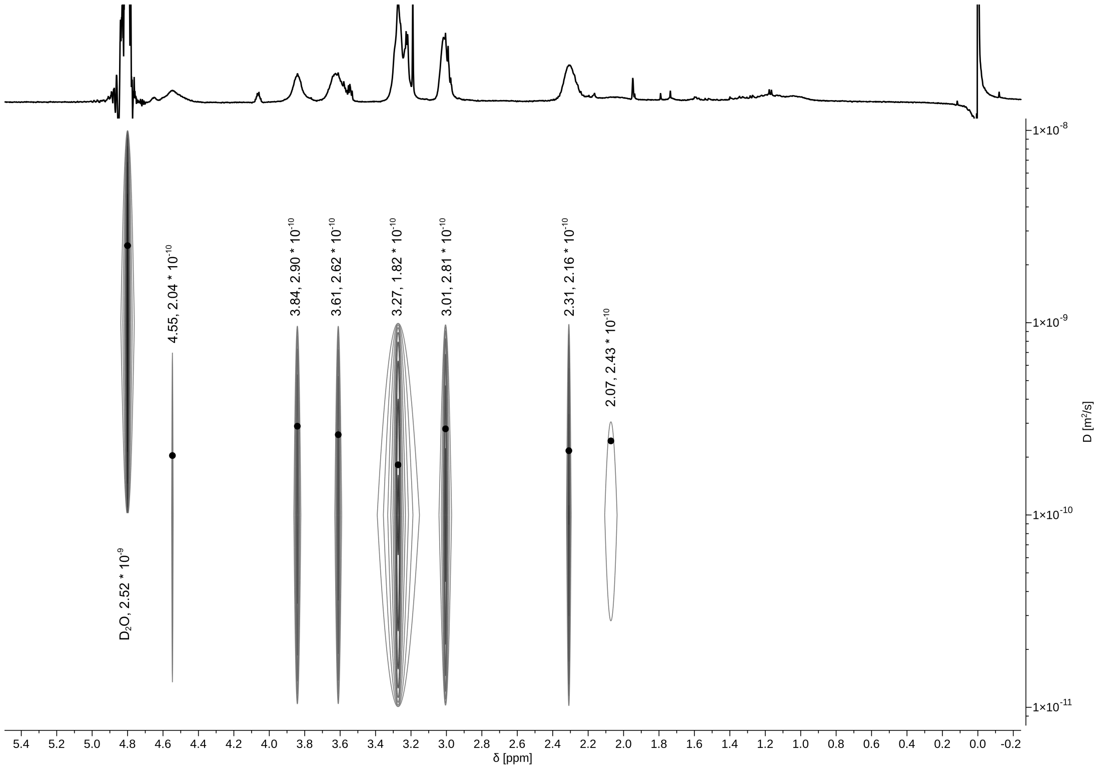

Figure SF24: DOSY NMR of poly([2-(methacryloyloxy)ethyl]dimethyl-(3-sulfopropyl)ammonium hydroxide) (PDMAPS) in D_2O.

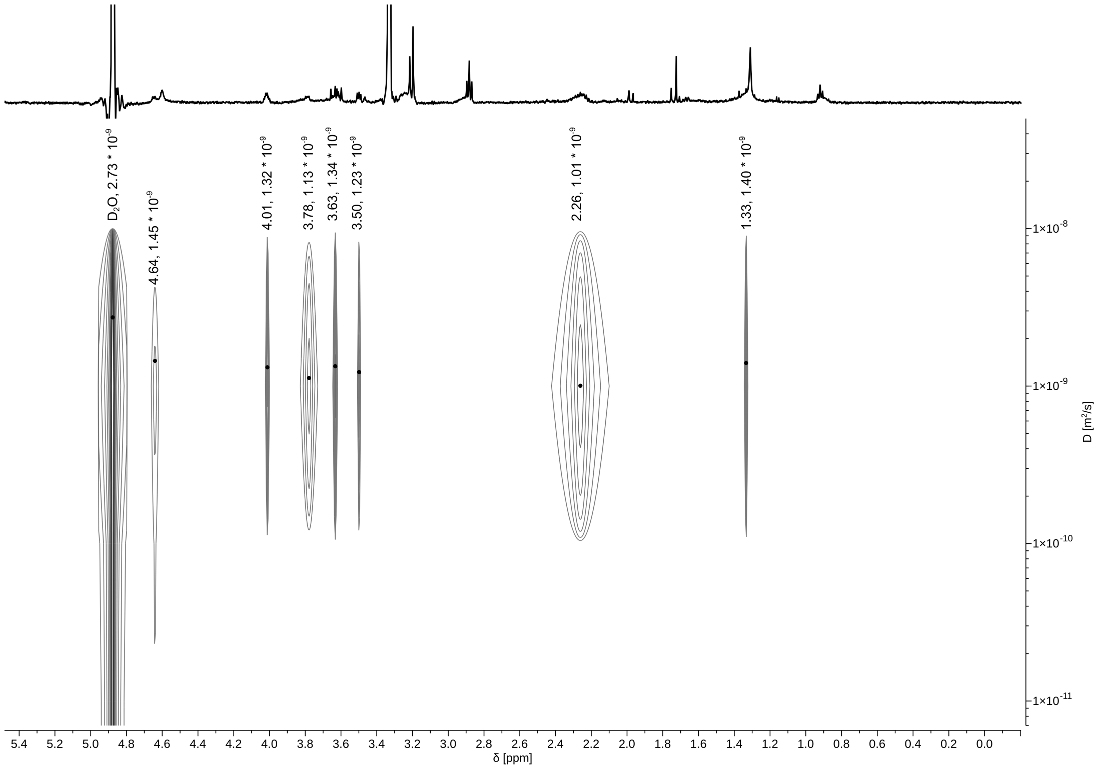

Figure SF25: DOSY NMR of poly([2-(methacryloyloxy)ethyl]dimethyl-(3-sulfopropyl)ammonium hydroxide) (PDMAPS) in MeOD.

S4.2 FT-IR

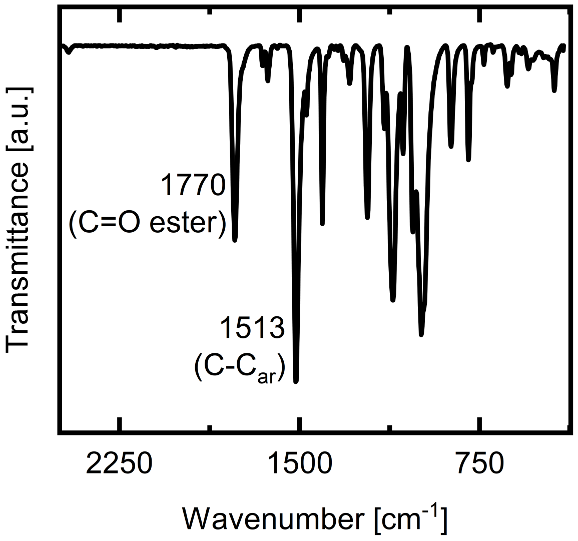

Figure SF26: FT-IR spectrum of pentalfluorophenyl acrylate (PFPA).

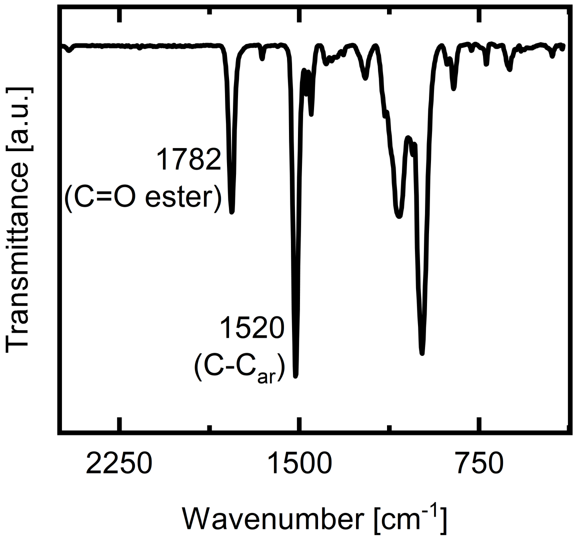

Figure SF27: FT-IR spectrum of poly(pentafluorophenyl acrylate) (PPFPA).

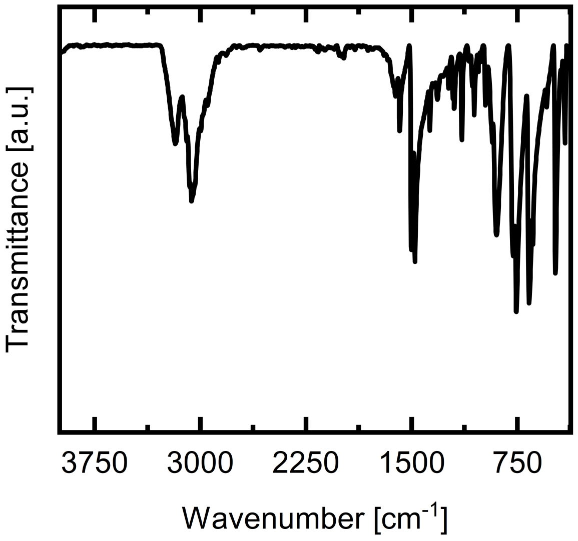

Figure SF28: FT-IR spectrum of 1-aminopyridinium idodide (1-API).

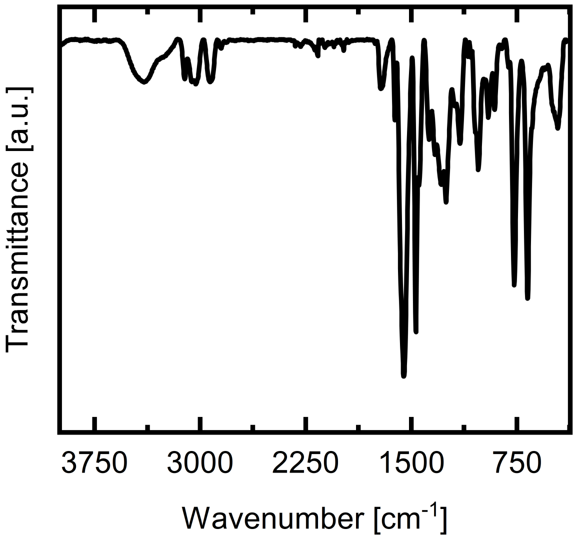

Figure SF29: FT-IR spectrum of poly(iminopyridinium ylide) (PIPY).

S4.3 Chromatography

Note: Acquisition of a SEC chromatogram for PMPC was not possible because of an incompatibility with the solvent-mixture.

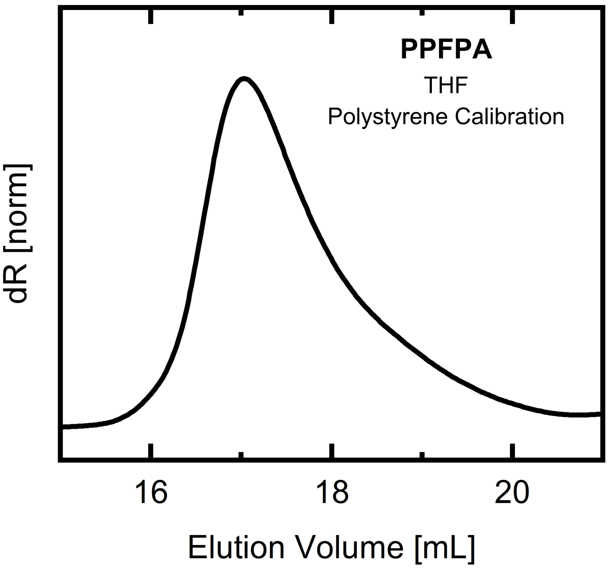

Figure SF30: SEC trace of PPFPA (THF, PS-standard). M_n = 8.2 kDa; Đ= 1.48.

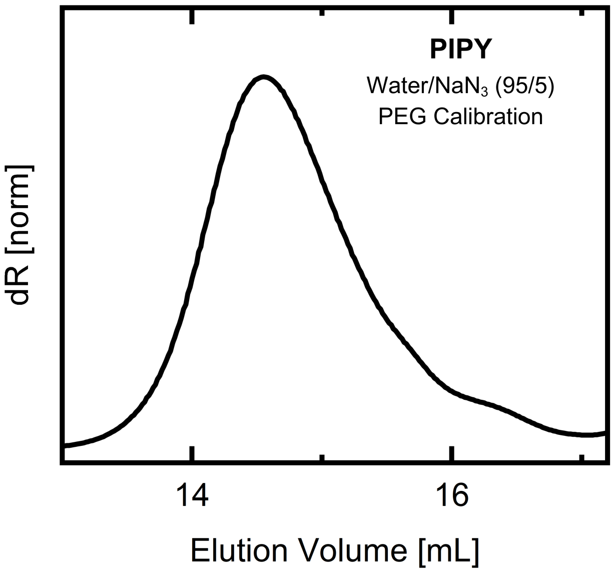

Figure SF31: SEC trace of PIPY (water/NaN_3, PEG-calibration/standard). M_n = 26.2 kDa; Đ= 1.38.

S4.4 Zeta-Potential

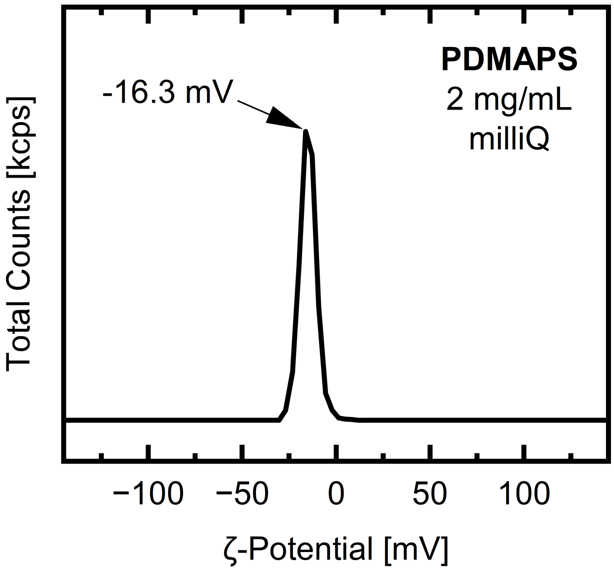

Figure SF32: Zeta-potential of poly([2-(methacryloyloxy)ethyl]dimethyl-(3-sulfopropyl)ammonium hydroxide) (PDMAPS) at 2 mg/mL (above the critical aggregation concentration (CAC)) in milliQ.

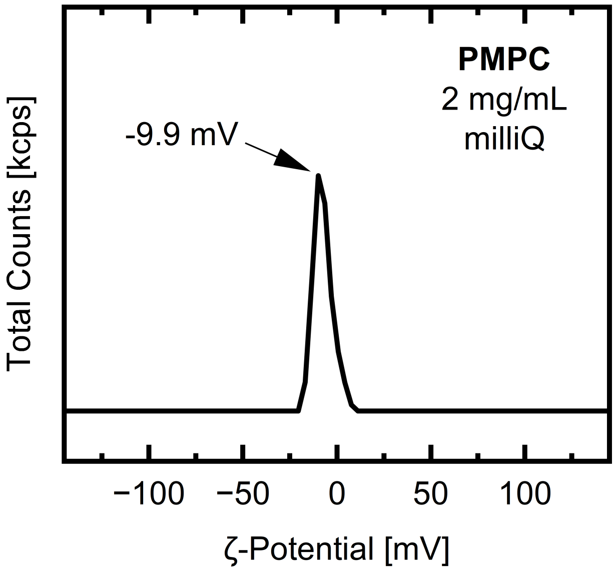

Figure SF33: Zeta-potential of poly(2-methacryloyloxyethyl phosphorylcholine) (PMPC) at 2 mg/mL (above the critical aggregation concentration (CAC)) in milliQ.

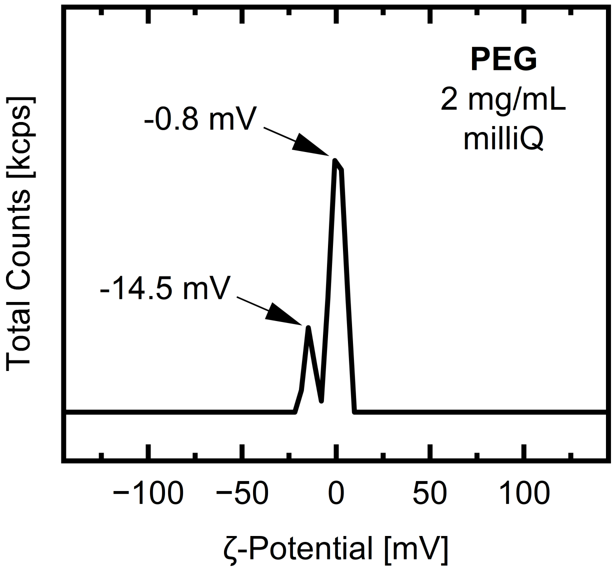

Figure SF34: Zeta-potential of poly(ethylene glycol) (PEG) at 2 mg/mL in milliQ. The double-peak structure is reproducible across several independent measurements and delivers a weighted average zeta-potential of -2.2 mV.

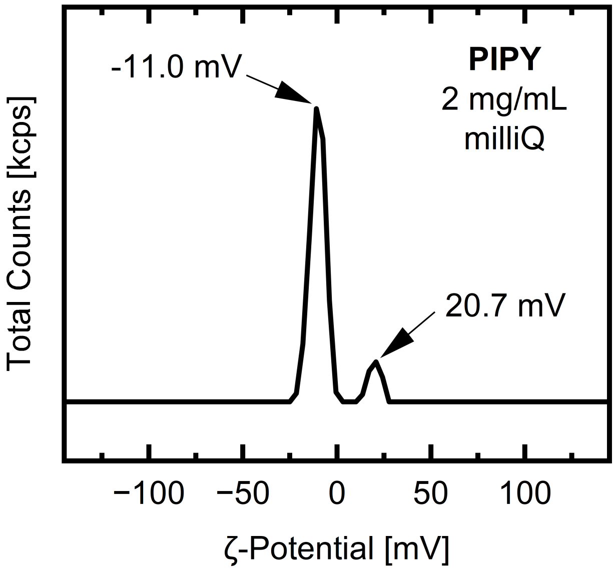

Figure SF35: Zeta-potential of poly(iminopyridinium ylide) (PIPY) at 2 mg/mL (above the critical aggregation concentration (CAC)) in milliQ. The double-peak structure is reproducible across several independent measurements and delivers a weighted average zeta-potential of -3.6 mV.

S4.5 Fluorescence Spectra of the Insulin Activity Assay

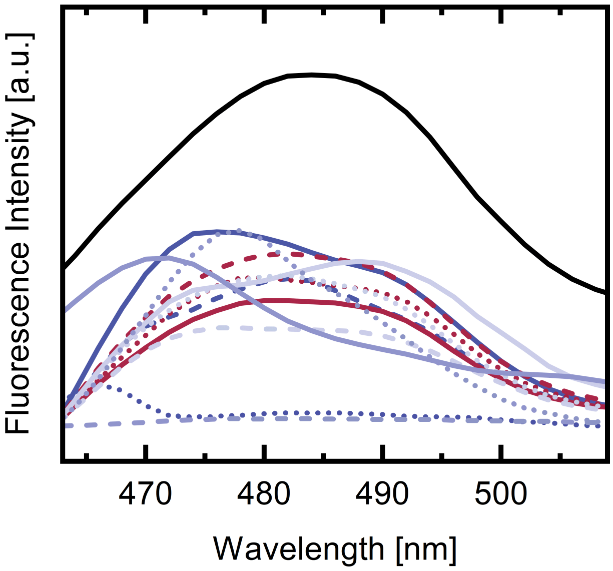

Figure SF36: Fluorescence emission of Thioflavin T in the presence of PEG, PIPY, PMPC, PDMAPS and no excipients (black) respectively. Full lines represent a concentration of 0.1 mg/mL, dashed lines of 0.5 mg/mL and dotted lines of 1.0 mg/mL.

S4.6 Circular Dichroism of BSA and PIPY

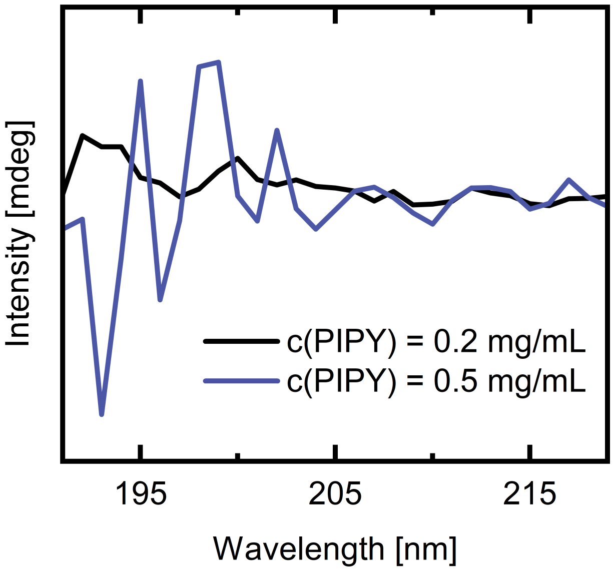

Figure SF37: Circular dichroism spectra of PIPY at 0.2 and 0.5 mg/mL respectively indicate no defined secondary structures of the polymer. Beyond the indicated concentrations, the spectrometer's detector was saturated and thus prevented the analysis of PIPY's presence on protein folding.

Acronyms

1-API 1-aminopyridinium idodide.

BSA bovine serum albumin.

CAC critical aggregation concentration.

DOSY diffusion-ordered spectroscopy.

ITC isothermal calorimetry.

LYZ lysozyme.

PDMAPS poly([2-(methacryloyloxy)ethyl]dimethyl-(3-sulfopropyl)ammonium hydroxide).

PEG poly(ethylene glycol).

PFPA pentalfluorophenyl acrylate.

PIPY poly(iminopyridinium ylide).

PMPC poly(2-methacryloyloxyethyl phosphorylcholine).

PPFPA poly(pentafluorophenyl acrylate).

References

(1) Menges, F. Spectragryph - optical spectroscopy software, 2022, http://www.effemm2.de/spectragryph/.

(2) Gürdap, S.; Bayram, N. N.; Isoglu, I. A.; Isoglu, S. D. Sulfobetaine-Based Homo- and Copolymers by RAFT: Cross-Linked Micelles and Aqueous Solution Properties. *ACS Applied Polymer Materials* **2022**, *4*, 6303–6311.

(3) Beattie, D. L.; Mykhaylyk, O. O.; Ryan, A. J.; Armes, S. P. Rational synthesis of novel biocompatible thermoresponsive block copolymer worm gels. *Soft Matter* **2021**, *17*, 5602–5612.
